# Scaling Transition of Active Turbulence from Two to Three Dimensions

**DOI:** 10.1002/advs.202402643

**Published:** 2024-08-13

**Authors:** Da Wei, Yaochen Yang, Xuefeng Wei, Ramin Golestanian, Ming Li, Fanlong Meng, Yi Peng

**Affiliations:** ^1^ Beijing National Laboratory for Condensed Matter Physics Institute of Physics Chinese Academy of Sciences Beijing 100190 China; ^2^ CAS Key Laboratory for Theoretical Physics Institute of Theoretical Physics Chinese Academy of Sciences Beijing 100190 China; ^3^ School of Physical Sciences University of Chinese Academy of Sciences 19A Yuquan Road Beijing 100049 China; ^4^ Wenzhou Institute University of Chinese Academy of Sciences Wenzhou Zhejiang 325000 China; ^5^ Max Planck Institute for Dynamics and Self‐Organization (MPIDS) D‐37077 Göttingen Germany; ^6^ Rudolf Peierls centre for Theoretical Physics University of Oxford Oxford OX1 3PU United Kingdom; ^7^ Songshan Lake Materials Laboratory Dongguan Guangdong 523808 China

**Keywords:** active matter, bacteria, collective motion, confinement, energy spectra

## Abstract

Turbulent flows are observed in low‐Reynolds active fluids, which display similar phenomenology to the classical inertial turbulence but are of a different nature. Understanding the dependence of this new type of turbulence on dimensionality is a fundamental challenge in non‐equilibrium physics. Real‐space structures and kinetic energy spectra of bacterial turbulence are experimentally measured from two to three dimensions. The turbulence shows three regimes separated by two critical confinement heights, resulting from the competition of bacterial length, vortex size and confinement height. Meanwhile, the kinetic energy spectra display distinct universal scaling laws in quasi‐2D and 3D regimes, independent of bacterial activity, length, and confinement height, whereas scaling exponents transition in two steps around the critical heights. The scaling behaviors are well captured by the hydrodynamic model we develop, which employs image systems to represent the effects of confining boundaries. The study suggests a framework for investigating the effect of dimensionality on non‐equilibrium self‐organized systems.

## Graphical abstract

The statistical properties of active turbulence are inherently dictated by dimensionality. How bacterial turbulence, the collective motion of dense swimming bacteria, transitions from two to three dimensions is elucidated. Combining experiments and theory, it is revealed that the transition takes two steps featured by scaling laws, and is governed by the competition among sizes of the bacteria, the vortex, and the confinement.

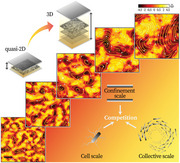

## 1 Introduction

Active fluids exhibit spontaneous turbulent flows at low Reynolds number (*Re*), which have been observed in bacterial suspensions,^[^ ^1^, ^2^, ^3^ ^]^ sperm swarms,^[^ ^4^ ^]^ mixtures of microtubules and motor proteins,^[^ ^5^, ^6^, ^7^ ^]^ epithelial cells,^[^ ^8^, ^9^ ^]^ and artificial motile colloids.^[^ ^10^ ^]^ These active fluids are constituted of a large number of self‐driven agents that inject kinetic energy individually into the systems. These agents generate active stress and drive the formation of turbulence. The internal‐driven and self‐organized nature distinguish this new class of turbulence from the classical ones. In this light, they are referred to as active turbulence.^[^ ^11^ ^]^

The behaviors of active turbulence largely depend on the confining boundaries and dimensionality. It has been shown that active turbulence bears different flow patterns in thin and thick samples.^[^ ^12^, ^13^ ^]^ The confinements alter the chaotic flows to directed ones by facilitating the coherent collective motion of active agents.^[^ ^6^, ^14^, ^15^, ^16^, ^17^, ^18^, ^19^ ^]^ Despite the consensus on the crucial role of dimensionality and confinement, how active turbulence evolves from 2D to 3D remains unexplored. For example, turbulences are realized by confining active fluid between two rigid walls separated by varying heights. Whether the turbulent flows are 2D, 3D, or none of them and how they evolve from 2D to 3D are unknown.

Following the seminal work by Kolmogorov,^[^ ^20^ ^]^ scaling behaviors of kinetic energy spectra have been widely used to reveal the underlying physics of complex flows. In many (but not all) classical turbulences, an inertial regime that exhibits the –5/3 scaling law predicted by Kolmogorov has been found, and this reveals a valuable common attribute between the turbulent flows in disparate systems. To emphasize its extensive applicability, this scaling law is often referred to as “universal.” For the various types of active turbulence, a universal (common) scaling law has long been sought, in the hope of uncovering a shared physical basis. However, the existence of such a law for active turbulences is still under debate. A phenomenological model, which is initially developed to for bacterial turbulence,^[^ ^12^, ^21^, ^22^ ^]^ predicts parameter‐dependent scaling laws^[^ ^23^ ^]^ and the scaling converges to –3/2 beyond a critical activity.^[^ ^24^ ^]^ Experimental verification of this model has remained incomplete. In contrast, models of active liquid crystals point to universal scaling^[^ ^25^, ^26^ ^]^ for 2D active nematic turbulence. Recent 2D experiments are in line with this view.^[^ ^27^ ^]^ Nevertheless, a cohesive understanding is still lacking.

Here, we combine experiments and theories to resolve how active turbulence evolves from 2D to 3D and whether it exhibits universal statistical properties. We experimentally characterize turbulent flows in bacterial suspensions by measuring spatial velocity correlations, structure functions, and kinetic energy spectra with varying confinement heights. As a result, we uncover that the turbulent flows undergo a two‐step transition at two critical heights. The two heights classify the turbulence into three regimes and emerge from the competition between system height and bacterial length, and that between the confinement height and vortex size. Our results show that bacterial turbulence follows universal scaling laws in the 2D and 3D limits, independent of cell activity and length, and the system height. A hydrodynamic model is developed to understand the universal scaling in the 2D and 3D limits, as well as the transition between them, which is consistent with our experiments.

## 2 Results

### 2.1 The Structures of Active Turbulence

**Figure 1:**
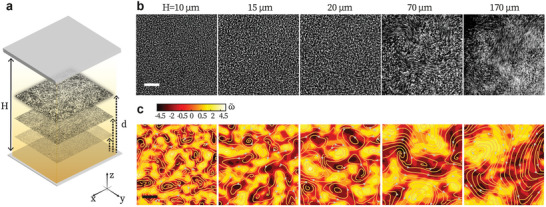
Bacterial turbulence in samples of increasing confinement size. a) Schematics of bacterial suspension confined between two parallel walls spaced by *H*. Bright field microscopy is performed at a distance *d* from the bottom wall. b,c) Typical flow patterns sampled at *d*_*c*_ = *H*/2 for different *H*. b) The time‐elapsed images. The elapsing time of each panel corresponds to ≈5m 〈*v*〉^−1^, where 〈*v*〉 is the mean flow field speed. c) Corresponding vorticity field and streamlines. $\tilde{\omega }$ represents the vorticity scaled by its mean absolute value over the field (〈|ω|〉). Scale bars: 20 m.

We employ *Escherichia coli* as our model system to study active turbulence (see Methods). Bacteria are suspended in a minimum motility buffer in which bacteria cannot grow further. To examine the effect of dimensionality, we inject bacterial suspensions into chambers confined in *z* −direction with different heights *H* (2‐400 m, dimensions in *xy*‐plane are 5–10 mm, Figure S1a, Supporting Information). The chamber is sealed to avoid external flows and oxygen gradient. The bacteria concentration *n* = 3.2 × 10^10^ cell mL^–1^, which corresponds to bacterial volume fraction ϕ≈6% (see Experimental Section). This concentration is higher than the critical concentrations of bacterial turbulence for all system heights. Under this concentration, the dynamics of turbulence is insensitive to both variation in bacterial activity and density. We choose this concentration also because bacteria interact with their neighbors mainly through hydrodynamic interactions,^[^ ^2^ ^]^ which aligns with hydrodynamic models we develop. We use video microscopy (30 fps) to image bacterial flow in *xy*‐plane at a distance (*d*) from the bottom of the chamber (**Figure** **1**a). We perform particle image velocimetry (PIV) to extract the velocity field v(r) and compute the vorticity field ω(r) (Figure 1b,c, Experimental Section).

**Figure 2:**
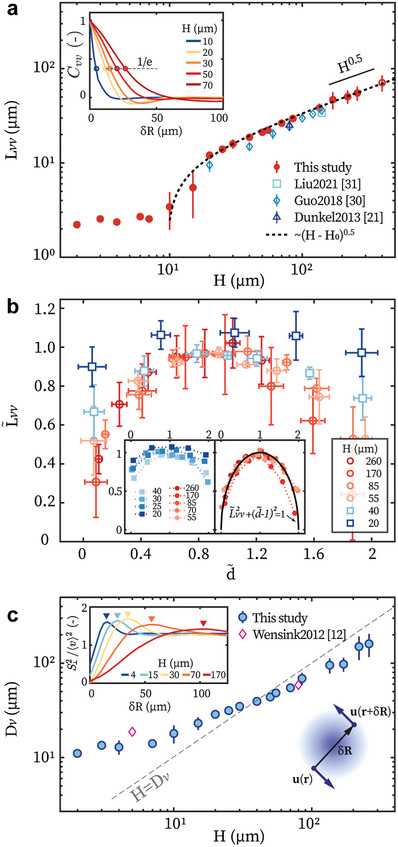
The velocity structures of bacterial turbulence. a) *L*_*vv*_ as a function of *H*. Data for *H* = 400m (hollow symbol) are exper imentally estimated, see Experimental Section. Inset: representative *C*_*vv*_ as a function of δ*R*; and *L*_*vv*_ is defined as where *C*_*vv*_(δ*R*) = 1/*e*. Dashed line: best fit with $L_{vv}=\sqrt{a(H-H_{0})}$.^[^ ^18^ ^]^ b) ${\tilde{L}}_{vv}=L_{vv}/\sqrt{a(H-H_{0})}$ as a function of $\tilde{d}=2d/H$. Left inset: measured in samples with *H* ⩽ 40 µm; right inset: with *H* > 40 µm. c) Effective vortex diameter *D*_*v*_ as a function of *H*. Inset: typical $S_{⊥}^{2}$; and *D*_*v*_ marks the peak position of $S_{⊥}^{2}$. Symbols and error bars in this figure represent mean ± standard deviation. Data in (a) and (c) are taken at *d*_*c*_ = *H*/2.

Bacterial turbulence exhibits distinctive flow patterns from 2D to 3D (Figure 1; Movies S1–S3, Supporting Information). To characterize the evolving patterns, we first calculate the spatial velocity correlation functions, $C_{vv}\left(\delta R\right)=\left\langle v\left(r\right)\cdot v\left(r+\delta R\right)\right\rangle /\left\langle v\left(r\right)\cdot v\left(r\right)\right\rangle$, where δ*R* is the magnitude of the spatial distance vector δR, and the angular brackets 〈 · 〉 represents averaging over all possible vector products of v within the flow field (including all angles of δR) and over the time of each recording (5 s). Representative *C*_*vv*_ measured at the central height *d*_*c*_ = *H*/2 in samples of different *H* are displayed in **Figure** **2**a inset. The correlation length *L*_*vv*_ is measured as the distance where *C*_*vv*_ drops to 1/*e*.^[^ ^27^, ^28^ ^]^ For a particular *H*, *L*_*vv*_ is robust against variations in bacterial activity after the onset of turbulence,^[^ ^22^, ^29^ ^]^ see also Figure S4 (Supporting Information). *L*_*vv*_ increases negligibly when *H* is below a critical height *H*_0_, whereas it soars up as $L_{vv}=\sqrt{a(H-H_{0})}$ above *H*_0_ (Figure 2a; *a* = 12.3 µm, *H*_0_ = 9.6 µm). Our results, which agree with previous experimental measurements,^[^ ^21^, ^30^, ^31^ ^]^ cover a wider range of the system heights. This allows us to resolve the critical length *H*_0_ in the 2D samples, and to discover the scaling *L*_*vv*_ ∝ *H* ^0.5^ in the 3D limit—which does not saturate up to *H* = 400 µm. To understand this scaling, we conduct stability analysis on active polar systems confined by two no‐slip walls, see Supporting Information. We find that the confining boundaries make the system deviate from the long‐wavelength instability predicted by the kinetic theory.^[^ ^32^, ^33^ ^]^ The wavelength corresponding to the fastest growth rate increases as *H* ^0.5^ in thick systems, which reflects the characteristic lengths of bacterial turbulence and underlies the observed trend in *L*_*vv*_(*H*).

At a certain *H*, *L*_*vv*_ varies symmetrically with *d* (Figure 1a) with respect to *d*_*c*_ = *H*/2 (Figure 2 and Figure S3, Supporting Information). We make *L*_*vv*_ and *d* dimensionless: ${\tilde{L}}_{vv}=L_{vv}/\sqrt{a(H-H_{0})}$ and $\tilde{d}=2d/H$ (Figure 2b). When *H* is slightly above *H*_0_, ${\tilde{L}}_{vv}$ depends weakly on $\tilde{d}$, e.g., when *H* = 20 m ${\tilde{L}}_{vv}$ varies less than 10% over the entire *z* domain (Figure 2b inset). As *H* increases, the dependence of ${\tilde{L}}_{vv}$ on $\tilde{d}$ becomes stronger (Figure 2b inset). Interestingly, above a critical height *H*_1_ ≈ 40 µm, ${\tilde{L}}_{vv}(\tilde{d}$) collapses to a master curve ${\tilde{L}}_{vv}^{2}+{\left(\tilde{d}-1\right)}^{2}=1$, which is *H*‐independent (Figure 2b insets). Such universal relation reveals scale‐invariant turbulence structures for *H* ⩾ *H*_1_.

We employ velocity structure functions to further resolve the structure of bacterial turbulence.^[^ ^12^, ^34^, ^35^ ^]^ They average the two‐point velocity difference δu=u(r+δR)−u(r) on orthogonal directions of δR over the flow domain: 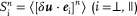 respectively. The maxima of $S_{⊥}^{2}$ correspond to an effective vortex size *D*_*v*_ (Figure 2c inset).^[^ ^34^ ^]^ Importantly, *D*_*v*_(*H*) intersects with *H* = *D*_*v*_ at *H*_1_ (Figure 2c), suggesting that *H*_1_ emerges from the interplay between the vortex size and the confinement, e.g., due to the suppression of *z*‐flows by the walls when *H* < *D*_*v*_. The near‐wall hydrodynamics is long known to lead rod‐shaped bacteria to swimming parallel to the wall,^[^ ^36^ ^]^ and confinement smaller than vortex sizes rectify bacterial flow from turbulent to coherent (i.e., with uniform direction).^[^ ^14^, ^15^ ^]^ To identify this, we measure flow velocity in all three dimensions simultaneously near the focal plane with an enhanced particle tracking velocimetry (Experimental Section; Figure S6, Supporting Information). A strong suppression of *z*‐flow is confirmed in thin samples, revealed by the large ratio of the *xy*‐velocity to the velocity in *z*, 〈*u*_*xy*_/*u*_*z*_〉. The suppression is relaxed after *H* ⩾ 40m ≈*H*_1_ ‐ as 〈*u*_*xy*_/*u*_*z*_〉 has reduced to the isotropic value $\sqrt{2}$ (Figure S6, Supporting Information). Altogether, by analyzing velocity correlations and structures of active turbulence in real space, we classify the turbulent flows into three regimes: (quasi‐) 2D (*H* ⩽ *H*_0_), 2D–3D crossover (*H*_0_ < *H* ⩽ *H*_1_), and 3D (*H* > *H*_1_). Note that, our data for the (quasi‐)2D regime (*H* ⩽ *H*_0_) are obtained from both 2D samples consisting of a single layer of swimming bacteria as well as from quasi‐2D samples containing a few layers of bacteria. For simplicity, we refer to both of them as 2D regimes and do not distinguish between these cases in the following analysis.

### 2.2 Kinetic Energy Spectra and Scaling Laws

**Figure 3:**
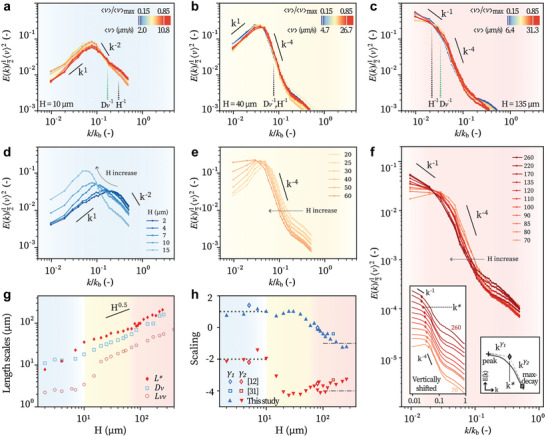
Kinetic energy spectra of bacterial turbulence in different regimes. Energy spectra for different activities (〈*v*〉) at representative heights: *H* ≈ *H*_0_ (a), *H* ≈ *H*_1_ (b), and *H* > *H*_1_ (c). 〈*v*〉_max_ is highest mean speed observed over multiple samples of the same height. The shown wavenumbers are scaled with *k*_*b*_ = 2π/*L*_*b*0_, where *L*_*b*0_ = 3 m is the bacterial length. Energy spectra evolving with *H* for *H* ≲ *H*_0_ (d), *H*_0_ ≲ *H* ≲ *H*_1_ (e), and *H* > *H*_1_ (f). Every spectrum is averaged over the activity range 〈*v*〉/〈*v*〉_max_ > 0.5. (g) Characteristic lengths extracted. L* = 2π/k*, with k* defined as shown in the schematics in f right inset. Left inset of (f): spectra vertically shifted for better visualization and with k* marked marked as diamonds. (h) Scaling exponents γ_1_ and γ_2_ as functions of *H*. Dotted and the dash‐dotted lines: theoretical prediction of (3). Blue, yellow, and red shading mark data obtained from 2D, 2D–3D crossover, and 3D regimes, respectively.

We next study how kinetic energy *E* is distributed on different length scales in active turbulence from 2D to 3D, by measuring the kinetic energy spectrum *E*(*k*) defined as *E* = ∫*E*(*k*)*dk* in the wavenumber(*k*)‐space. *E*(*k*) is computed as $\int_{k=k}\frac{1}{2}u\left(k\right)\cdot u^{*}\left(k\right)dk$, with u(k) the flow field represented in Fourier space. This definition of *E*(*k*) satisfies Wiener–Kinchine theorem (WKT): *E*(*k*) correlates with the Fourier transformation of the *C*_*vv*_ as $F\left(C_{vv}\right)∼E\left(k\right)/k$. *E*(*k*) is reported for each height over multiple samples at varying bacterial activities. At a specific *H*, the dimensionless energy spectra $E\left(k\right)/\frac{1}{2}{\left\langle u\right\rangle }^{2}$ are independent of the activity (**Figure** **3**a–c), showing the same scaling behaviors. Therefore, we report the dimensionless spectrum averaged over samples and activities for a certain *H* (Figure 3d–f).

In 2D (*H* ⩽ *H*_0_), the energy spectrum is featured by two scaling regimes:*E*(*k*) ≈ *k* at low‐*k* (large length scales) and ≈ *k* ^−2^ at high‐*k* (small length scales) (Figure 3a,d). Note that the rising tail at *k* ≳ 0.3*k*_*b*_ results from noise in velocimetry and microscopy in thicker samples. These spectra shift to lower *k* (Figure 3d‐f) as *H* increases, which can be measured by the turning point k* between two regimes of different scaling exponents (Figure 3f right inset). The corresponding lengths L* = 2π/k* are displayed in Figure 3g. They both evolves as *H* ^0.5^ in the 3D limit, same as *L*_*vv*_ and *D*_*v*_. All these three quantities reflect the averaged length scales of unstable modes in bacterial turbulence at varying *H*, yet in different definitions or averaging methods.

The scaling laws transition from 2D to 3D in two steps: the high‐*k* scaling exponent γ_2_ drops first from –2 to –4 at *H*_0_, and then the low‐*k* exponent γ_1_ changes from +1 to –1 when *H* > *H*_1_ (Figure 3h). The previous measurements on energy spectra are well in line with our results.^[^ ^12^, ^31^ ^]^ Altogether, from the kinetic energy spectra we obtain the same statistical properties of active turbulence: the *H* ^0.5^ scaling of vortex size (Figures 3g and 2a) and the two critical heights *H*_0_ and *H*_1_ (Figures 3h and 2a,b).

### 2.3 The Effect of Cell Length on Active Turbulence

**Figure 4:**
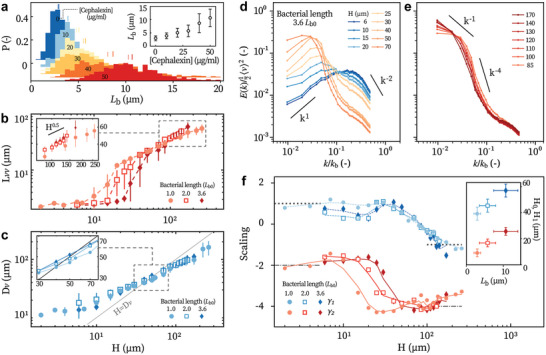
Effect of bacterial length on the turbulence a) The distributions of cell lengths under different concentrations of cephalexin [Cephalexin]. Dotted and solid short lines respectively mark the median and mean values. Inset: mean value of *L*_*b*_ for different [Cephalexin]. Error bars represent standard deviations. b) *L*_*vv*_ as a function of *H* for different *L*_*b*_. Inset: zoom in of the marked region. c) *D*_*v*_ as a function of *H* for different *L*_*b*_. d,e) Energy spectra obtained with the most elongated cells (3.6*L*_*b*0_). The blue‐yellow transition corresponds to *H*_0_. f) Spectral scaling γ_1_ and γ_2_ obtained with elongated bacteria. Black dotted and dash‐dotted lines: model predictions. Inset: *H*_0_ and *H*_1_ for different *L*_*b*_. Error bars show standard deviations of *L*_*b*_ (*x*‐axis) and uncertainties in measuring *H* (*y*‐axis). All measurements are obtained at *d*_*c*_ = *H*/2 with ϕ = 0.06.

Besides the system height and the vortex size, the cell length *L*_*b*_ is another fundamental length scale of the active turbulence system. To clarify how *L*_*b*_ affects the turbulent behaviors, we vary bacterial length by adding cephalexin, an antibiotics that prohibits cell division^[^ ^28^, ^37^ ^]^ (Experimental Section). *L*_*b*_ increases monotonically with the cephalexin concentration [Cephalexin], and meanwhile no obvious changes are found in the size distributions (**Figure** **4**a and Figure S2, Supporting Information) and the motility of a bacterium.^[^ ^28^, ^37^ ^]^ We experiment with bacteria whose mean length 〈*L*_*b*_〉 ≈ 2.0*L*_*b*0_ (5.8 m, [Cephalexin] = 30 g mL^–1^) and 3.6*L*_*b*0_ (10.7 m, [Cephalexin] = 50 µg mL^–1^) at the same bacterial volume fraction (6%).

Figure 4 shows the correlation length, vortex size, and energy spectra for different bacterial lengths. Varying *L*_*b*_ does not alter the *H* ^0.5^ scaling of *D*_*v*_ or *L*_*vv*_ (Figure 4b‐c). Energy spectra measured with elongated cells show the same scaling exponents in low‐*k* and high‐*k* regimes, with (γ_1_, γ_2_) = (+ 1, −2) in 2D and (–1, –4) in 3D, respectively (Figure 4d,e). This supports the universality of the scaling laws in bacterial turbulence. However, the critical heights increase for longer bacteria. *H*_0_ follows an empirical relation *H*_0_ = 2.1*L*_*b*_ + 2.3 µm and *H*_1_ increases similarly with *L*_*b*_ (Figure 4b,f).

Additionally, *H*_0_ and *H*_1_ measured in the representation of velocity structures (*L*_*vv*_ and *D*_*v*_) and in the representation of energy spectra display excellent equivalence (Figure S5, Supporting Information).

### 2.4 Hydrodynamic Theory and Universal Scaling

**Figure 5:**
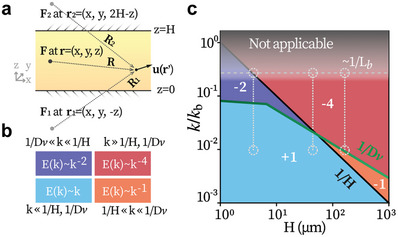
Scaling regimes predicted by the hydrodynamic model. a) Scheme of the hydrodynamic model, where boundary effects are approximated by two images. b) Asymptotic scaling behaviors results from the competition among *k*, *H* ^−1^, and $D_{v}^{-1}$. c) Predicted scaling regimes for the bacterial turbulence. The green lines represent the *D*_*v*_ − *H* relation. Each regime is marked by its scaling exponent. Vertical dashed lines: (from left to right) measured ranges corresponding to Figure 3a–c, respectively.

To understand the scaling behaviors of 2D and 3D active turbulence, we develop a hydrodynamic model for active fluids confined by two solid boundaries. The model considers the active fluid as a continuum force field, and represents the effects of the upper and the lower walls by two image systems. A Green function with the first two image reflections is employed to take account of the hydrodynamic screening effects of the two walls,^[^ ^38^, ^39^ ^]^ see **Figure** **5**a and Supporting Information for more details. The approximation is taken with the fact that no‐slip boundary conditions seem not satisfied at the scale of the bacterial length (see Figure 2b) in the 2D case. For strict no‐slip boundary conditions, one should use the Green function proposed by Liron with the summation of the infinite number of images.^[^ ^40^ ^]^ The energy spectrum *E*(*k*) written in the form of the tensor is:

(1) $$E\left(k\right)∼k\langle |\hat{u}\left(k\right){|}^{2}\rangle =k{\bar{G}}_{\alpha \beta }{\bar{G}}_{\alpha \gamma }\left\langle F_{\beta }F_{\gamma }^{*}\right\rangle$$

where ${\bar{G}}_{\alpha \beta }$ (α, β, γ = *x*, *y*) is the Green function in Fourier representation, and *F* is the active force density. The asymptotic behavior of ${\bar{G}}_{\alpha \beta }$ is:

(2) $${\bar{G}}_{\alpha \beta }∼\left\{\begin{array}{cc} \left(\delta_{\alpha \beta }-\frac{k_{\alpha }k_{\beta }}{2k^{2}}\right)\cdot \frac{-H}{k}, & kH\ll 1;\\ \left(\delta_{\alpha \beta }-\frac{k_{\alpha }k_{\beta }}{k^{2}}\right)\cdot \frac{2}{k^{2}}, & kH\gg 1 \end{array}\right.$$

Hence, correspondingly, *E*(*k*) behaves as:

(3) $$E\left(k\right)∼\left\{\begin{array}{cc} \frac{3H^{2}\eta^{2}k}{4}\left\langle \omega^{2}\left(k\right)\right\rangle +\frac{H^{2}}{4k}\left\langle F^{2}\left(k\right)\right\rangle , & kH\ll 1;\\ \frac{\eta^{2}}{k}\left\langle \omega^{2}\left(k\right)\right\rangle , & kH\gg 1 \end{array}\right.$$

Here, η is the shear viscosity and ω(k) the vorticity field in Fourier space. Furthermore, the scaling behavior of $\left\langle \omega^{2}\left(k\right)\right\rangle$ is governed by the competition between *k* and the wavenumber corresponding to the vortex size ($D_{v}^{-1}$). Under the assumptions that the active vortices are uncorrelated and exponentially distributed in size (Figure S8, Supporting Information), $\left\langle \omega^{2}\left(k\right)\right\rangle$ scales respectively as *k* ^0^ and *k* ^−3^ for *kD*_*v*_ ≪ 1 and *kD*_*v*_ ≫ 1.^[^ ^25^ ^]^ Altogether, in the limit *F* ≪ η*k*ω, the interplay among *k*, *H* ^−1^, and $D_{v}^{-1}$, give rise to four different scaling regimes, see Figure 5b. We implement the empirical relation between *D*_*v*_ and *H* from the experiments (Figure 2c) and display the predicted scaling regimes for our system in Figure 5c. It is worth noting that the cell length *L*_*b*_ sets the length scale below which the continuum model is not applicable.

The *k* ^1^‐ and *k* ^−4^‐scaling laws are revealed to be universal, and they govern the kinetic energy distribution on the large and small scales, respectively. On large scales ($k\ll H^{-1},D_{v}^{-1}$), the growth of long‐wavelength modes are suppressed by the presence of two confining boundaries, and such size selection underpins the *k* ^1^‐scaling. On the small scales ($k\gg H^{-1},D_{v}^{-1}$), the *k* ^−4^‐scaling marks the energy dissipation within vortices, which is dominated by viscosity.^[^ ^11^ ^]^ Meanwhile, *k* ^−2^ and *k* ^−1^ represent two mutually exclusive scaling laws, which govern the transitional regimes in the *k*‐space for 2D and 3D samples respectively. The *k* ^−2^‐scaling governs the regime $D_{v}^{-1}\ll k\ll H^{-1}$ and becomes evident in the 2D limit ($H^{-1}\gg k,D_{v}^{-1}$). On the other hand, the *k* ^−1^‐scaling governs $H^{-1}\ll k\ll D_{v}^{-1}$ and emerges in the 3D limit ($H^{-1}\ll k,D_{v}^{-1}$). In all, our model supports the existence of universal scaling for active turbulence in both the 2D and 3D limits.

Consistently, our experimental results agree with the predicted universal scaling. In 3D samples, the measured scaling (γ_1_, γ_2_) = (−1, −4) (Figure 3c), and *k* ^1^‐scaling are observed at small wavenumbers (Figure S7, Supporting Information). These all align perfectly with the prediction, see the right vertical dashed line in Figure 5c. For samples in the 2D‐3D crossover regime, e.g., around *H* = *H*_1_, the kinetic energy spectrum is predicted to evolve from *k* ^1^‐scaling to *k* ^−4^‐scaling with no transitional scaling (middle vertical line in Figure 5c). It is exactly what we observe (γ_1_, γ_2_) = (1, −4) around *H* = *H*_1_, see Figure 3b,e. Lastly, in the 2D limit, only the *k* ^1^‐ and *k* ^−2^‐scaling are observed (Figure 3a) because the predicted *k* ^−4^‐scaling only exists on the scales smaller than the bacterial size. On such scales, the bacterial suspensions cannot be regarded as a continuum and thus the model becomes not applicable, as is shown by the gray shaded area in Figure 5c.

## 3 Discussion

In summary, we have observed that bacterial turbulence exhibits a two‐step transition from 2D to 3D: spectral scaling governing the small length scales changes at *H*_0_ and a characteristic *k* ^−1^ scaling for 3D turbulence emerges after *H*_1_. The two critical heights are consistently found in evolution of real‐space flow structures. *H*_0_ results from the interplay of the confinement height *H* and bacterial length, whereas *H*_1_ reflects the competition between the system height and the vortex size. Experimental measurements indicate the scaling behaviors of bacterial turbulence in the 2D and 3D limits to be universal ‐ which are independent of bacterial activity, bacterial length, and system height. Note that the wavenumber ranges to obtain scaling exponents are narrow because bacterial length, vortex size and the confinement height cannot be orders‐of‐magnitude different. Similar difficulties also exist in previous experiments of active turbulence.^[^ ^12^, ^27^ ^]^ The universal scaling laws are confirmed by a hydrodynamic model considering active fluids confined between two solid walls. This model predicts universal *k* ^1^ and *k* ^−4^ scaling on large and small length scales, respectively, and *k* ^−2^ and *k* ^−1^ on intermediate length scales in 2D and 3D, respectively. Lastly, scale‐invariant flow structures, *H*‐independent ${\tilde{L}}_{vv}(\tilde{d}$) and a characteristic *H* ^0.5^‐dependence of vortex size, are uncovered experimentally for 3D bacterial turbulence. The *H* ^0.5^‐scaling is derived using stability analysis in confined active polar systems.

Our hydrodynamic model considers active fluids as a continuum force field and use image systems to capture the hydrodynamic screening effect of the confining boundaries as the leading‐order approximation. Here we do not consider the non‐uniformity in the density distribution, which could be important in presence of giant density fluctuations^[^ ^41^, ^42^ ^]^ and motility‐induced phase separation (MIPS).^[^ ^43^ ^]^ Also, the inner structure of the vortex is largely simplified in the sense that the continuum assumption ignores irregular shapes and complex kinetics of realistic vortices at small scales (high‐*k*).^[^ ^44^ ^]^ Nevertheless, the model still captures our experimental findings excellently, showing that the details above have only minor effects in determining the scaling properties of active turbulence. One possible reason is that bacterial turbulence is dominated by hydrodynamic interaction,^[^ ^2^ ^]^ which suppresses the density fluctuation and MIPS.^[^ ^43^ ^]^ In addition, our experiments confirm that the exponential vortex size distribution, which applies for active nematic turbulence,^[^ ^25^, ^27^ ^]^ is also a good approximation in bacterial turbulence. Hence, we believe our theoretical model captures the essential elements of scaling behaviors of active turbulence from 2D to 3D. The model can be applied in hydrodynamic‐dominated active systems where the vortex‐vortex interaction is negligible and MIPS is absent.

The conclusions of our experiments and theories show wide applicability. The energy spectra and vortex structures reported by other experiments that employ different bacteria species and/or are conducted at higher concentrations^[^ ^12^, ^31^ ^]^ are consistent with both our experimental results and theoretical predictions. Even in dense suspensions of mammalian sperms where the active agents are an order of magnitude larger than bacteria and are of a distinct propulsion mechanism, the energy spectrum still displays *k* ^−4^‐scaling when *k* ≫ 1/*H*, 1/*D*_*v*_, in line with our results.^[^ ^4^ ^]^ It is noteworthy that the existence of the two‐step transition and four scaling regimes does not rely on a specific *D*_*v*_ − *H* relation but only requires that *D*_*v*_ increases with *H* slower than linearly in the 3D limit (so that 1/*H* and 1/*D*_*v*_(*H*) can intersect). Therefore, similar scaling behaviors are expected in other active turbulence systems, which exhibit either *H* ^0.5^^[^ ^16^, ^18^, ^19^, ^45^ ^]^ or slower increase^[^ ^46^ ^]^ of correlation length in active nematics.

Besides hydrodynamic‐interaction‐dominated bacterial turbulence confined between two solid surfaces, the collective motion of active matter systems can be dominated by other interactions, like short‐ranged steric interaction^[^ ^47^ ^]^ and long‐ranged electromagnetic interactions.^[^ ^48^, ^49^ ^]^ The confining boundary could be liquid‐liquid interfaces^[^ ^27^ ^]^ or liquid–air interfaces,^[^ ^50^ ^]^ which provide distinct couplings within active fluids. The self‐organization of active entities could be manipulated by external fields.^[^ ^51^ ^]^ How these factors affect the universal scaling is a question worth further exploring.

## 4 Experimental Section

### Fluidic Chamber

Fluidic chambers were made with glass slides and coverslips as shown in Figure S1a (Supporting Information). After being filled with bacteria suspension, chambers were sealed with UV‐cured glue (NOA63, Norland Optics Inc.). To tune the chamber heights, polystyrene beads were used as spacers for chamber height *H* < 10 m, and different tapes for chambers of *H* ≳ 7 m. Besides chambers of uniform height, sloped chambers were made using tapes of different heights (*H*′ ≠ *H*″) and were calibrated per piece. They allowed fine tune over *H* and high‐throughput experimentation. In such chambers, the bottom and top plates are approximately parallel (incline angle <0.4°). Turbulent statistics measured in those two types of chambers are same, see Figure S1b (Supporting Information).

### Bacterial Suspension

A wild‐type *E.coli* strain (BW25113) was employed in the experiment. To extend the swimming period in closed chambers, a light‐driven transmembrane proton pump, proteorhodopsin (PR), was inserted into the strain.^[^ ^52^ ^]^ A typical cell body has a length *L*_*b*_ of 3 m and a width *W*_*b*_ of 0.8 m. First, the bacteria was inoculated in Terrific Broth (TB) medium and incubated overnight (12–16 h) at 37 °C. The saturated suspension was diluted for 100 times with fresh TB medium and then was further cultured at 30 °C for 6–8 h. Bacteria were harvested and centrifuged at 800 − 1000 *g* for 5 min. The bacteria were resuspended in motility buffer, containing 6.2 mM K_2_HPO_4_, 3.8 mM KH_2_PO_4_, 67 mM NaCl, 0.1 mM EDTA, 4.5 × 10^−3^% (v/v) TWEEN20, and 0.5% (w/v) glucose. The final body volume fraction of bacteria was ϕ ≈ 0.06 (number density *n* ≈ 3.2 × 10^10^ cells mL^–1^, $\phi =nL_{b}W_{b}^{2}$).

### Cephalexin Treatment

Bacterial length was tuned by inducing cephalexin (Solarbio Inc.) of different concentrations ([Cephalexin]) at 3.5 h after 3.5 h into the second stage of culturing. The lengths for N ≈ 1000 cell were measured at each [Cephalexin]. The length polydispersity was unvaried, see Figure S2 (Supporting Information). Additionally, bacterial motility was not affected by the cephalexin treatment. The mean swimming speed of single cells were measured in H = 8 m samples (N ≈ 20 for each concentration) and they remained constant in the tested antibiotic concentrations, in line Ref. [37, 53]. In the series of experiments involving cephalexin, the cell body volume fraction was maintained at ϕ ≈ 0.06.

### Flow Field Extraction

Bright field microscopy was performed with an inverted microscope (Nikon Ti2e). A 40 × objective was employed primarily for measurement and a 10 × objective was used to obtain a large field of view, see Figure S7 (Supporting Information). Videos were recorded with an sCMOS camera at 30 fps (PCO.edge). A typical measurement lasts for 5 s. For each sample of uniform height, ≈20 measurements were taken; while in the sloped chamber, ≈8 measurements were performed at each height (location). Recorded videos were analyzed with particle image velocimetry (matlab PIVLab2.37^[^ ^54^ ^]^). Windows of 16 × 16 pixels with 50% overlap were used for convolution.

### Measuring Correlation Length for ***H* =**400 m

For the thickest samples measured (*H* = 400 µm , empty red marker at the right extreme of Figure 2a), flow patterns at *d*_*c*_ = *H*/2 were too obscured to extract. The presented values were asymptotic estimations by extrapolating the trend measured with ϕ = 0.03 from *d* = 0 − 120 m, and ϕ = 0.06 from *d* = 0 −70 m.

### Data Pooling and Averaging

For each confinement height *H*, the highest possible mean turbulence speed 〈*v*〉_max_ was employed to benchmark the activity level of a particular bacterial suspension, see Figure 3a–c. 〈*v*〉_max_ was obtained from measurements over multiple samples, which were prepared following the same protocol. The energy spectrum of a particular *H* displayed in Figures 3d–f and 4 was averaged over 〈*v*〉/〈*v*〉_max_ >0.5. Although *L*_*vv*_ and *D*_*v*_ were found to depend negligibly on 〈*v*〉 after the onset of turbulence (Figure S4a, Supporting Information), same as reported previously.^[^ ^21^, ^22^, ^29^ ^]^ However, for consistency, the same criterion on activity was applied for *L*_*vv*_ and *D*_*v*_. In measuring *L*_*vv*_ as a function of *d*, no criteria were set for bacterial activity as 〈*v*〉 varies with *d*. The raw measurements of *L*_*vv*_(*d*) are displayed in Figure S3 (Supporting Information).

### Enhanced Particle Tracking Velocimetry

An enhanced particle tracking velocimetry (PTV) technique was employed to measure the *z*‐component of speed, *u*_*z*_, near the focal plane. The images of beads that were slightly out of focus, by 1) the fitting quality to a Laplacian‐of‐Gaussian (LoG) kernel and 2) total pixel intensity of the beads' interior. These metrics were extracted by TrackMate plugin of ImageJ^[^ ^55^ ^]^ and together calibrate a bead's *z* position. In this way, all three coordinates of the beads were obtained within a slice of ≈2*a* thickness around the focal plane (*a* = 6m is the bead diameter). 2 × 10^3^ − 1 × 10^4^ tracks were collected per sample height, with which velocity statistics were performed. See the detailed method in Supporting Information and Figure S6 (Supporting Information).

## Conflict of Interest

The authors declare no conflict of interest.

## Author Contributions

Y.P. and F.M. conceived the project. D.W. performed the experiments and analyzed the data. F.M., R.G., Y.Y., and X.W. conducted the theoretical modeling and calculations. D.W., Y.P., and F.M. wrote the paper with input from M.L. and R.G. All authors discussed the results and reviewed the manuscript.

## Supporting information

Supporting Information

Movie 1

Movie 2

Movie 3

## Data Availability

The data that support the findings of this study are available from the corresponding author upon reasonable request.
