# Supplementary material for: Scaling Transition of Active Turbulence from Two to Three Dimensions
Source: Adv Sci (Weinh). 2024 Aug 13;11(38):2402643. doi: 10.1002/advs.202402643 (PMC11481389; doi:10.1002/advs.202402643)
Supplement: Supplementary file 1 — Supporting Information [file ADVS-11-2402643-s004.pdf]

ADVANCED SCIENCE
Open Access

Supporting Information

for *Adv. Sci.*, DOI 10.1002/advs.202402643

Scaling Transition of Active Turbulence from Two to Three Dimensions

Da Wei, Yaochen Yang, Xuefeng Wei, Ramin Golestanian, Ming Li, Fanlong Meng and Yi Peng**

Supporting Information for *Scaling transition of active turbulence from two to three dimensions*

Da Wei[1], Yaochen Yang[2,3], Xuefeng Wei[2,3,4],
Ramin Golestanian[5,6], Ming Li[1,7], Fanlong Meng[2,3,4]⋆, Yi Peng[1,3]†

[1]Beijing National Laboratory for Condensed Matter Physics, Institute of Physics, Chinese Academy of Sciences, Beijing 100190, China
[2]CAS Key Laboratory for Theoretical Physics, Institute of Theoretical Physics, Chinese Academy of Sciences, Beijing 100190, China
[3]School of Physical Sciences, University of Chinese Academy of Sciences, 19A Yuquan Road, Beijing 100049, China
[4]Wenzhou Institute, University of Chinese Academy of Sciences, Wenzhou, Zhejiang 325000, China
[5]Max Planck Institute for Dynamics and Self-Organization (MPIDS), D-37077 Göttingen, Germany
[6]Rudolf Peierls center for Theoretical Physics, University of Oxford, Oxford OX1 3PU, United Kingdom
[7]Songshan Lake Materials Laboratory, Dongguan, Guangdong 523808, China
†Corresponding author. Email: pengy@iphy.ac.cn;
⋆Corresponding author. Email: fanlong.meng@itp.ac.cn.

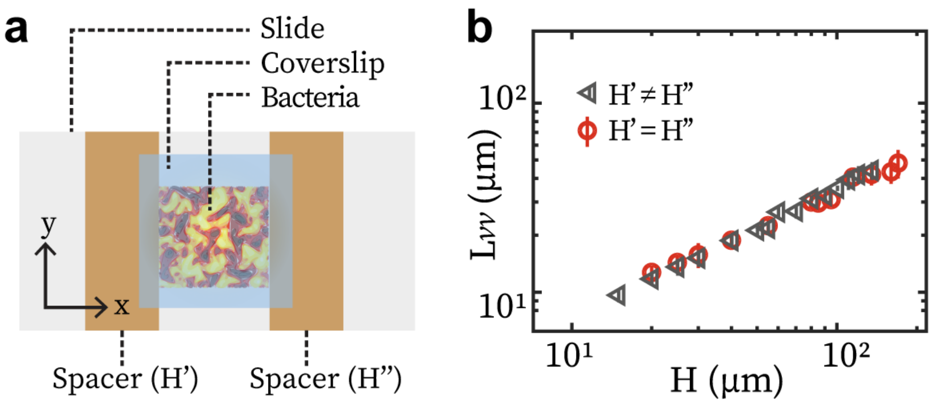

Figure S1: **Data taken in different fluidic chambers.** (a) Schematics of a chamber. (b) Velocity correlation length measured in the wedged chambers ($H' \neq H''$) and uniform-height chambers($H' = H''$). Symbols and error bars represent mean and one standard deviation respectively.

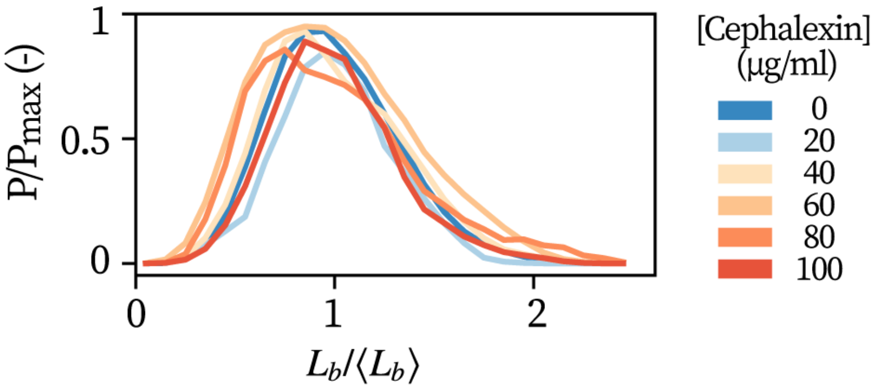

Figure S2: **Normalized cell length distribution after cephalexin treatment.** Distributions of scaled cell length $L_b/\langle L_b \rangle$ are further normalized by their respective maxima.

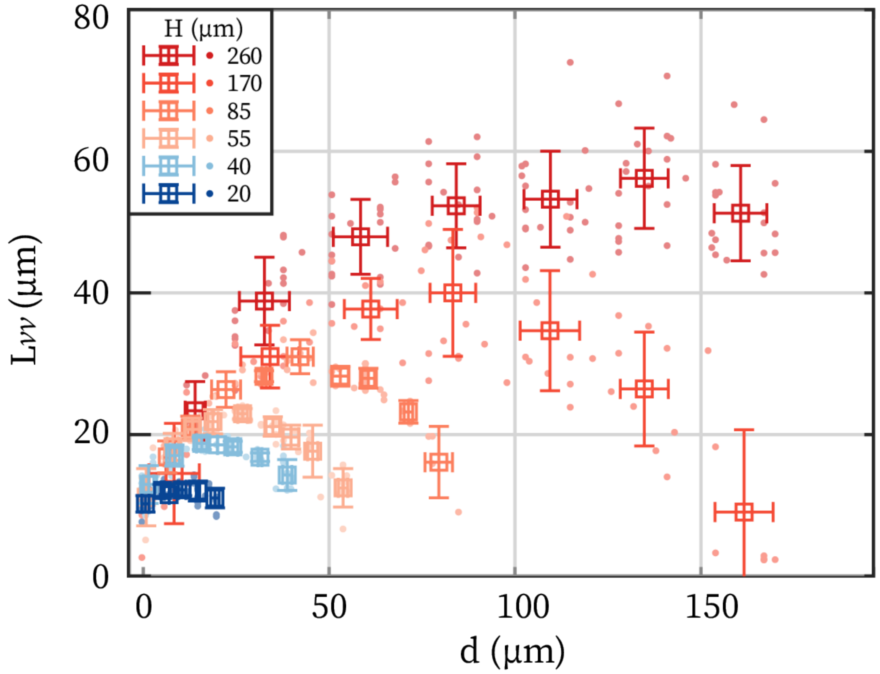

Figure S3: **Variation of the correlation length in z.** Measurements of L_{vv} as a function of d (N=3-5 samples) are plotted as dots. Symbols with error bars represent the mean and standard deviation of the binned data.

S1 Spatial structure of turbulence is independent on bacterial density and activity

Bacteria are known to accumulate near no-slip boundaries (*1*). Such accumulation may induce number density variation along the system's z axis. If the characteristic lengths of turbulence (e.g., L_{vv} and D_v) depend on bacterial number density, then the near-wall accumulation will induce uncertainties in the reported data, for example, in L_{vv}'s dependence on d (Fig. 2, main text). However, we show that bacterial number density matters negligibly when it is sufficient for turbulence to emerge. Both L_{vv} and D_v remain almost constant after the bacterial volume ratio ϕ exceeds ~3%, see Fig. S4b and c respectively. The presented data are measured at $d_c = H/2$, and we confirm that the trends are qualitatively the same for other heights (d).

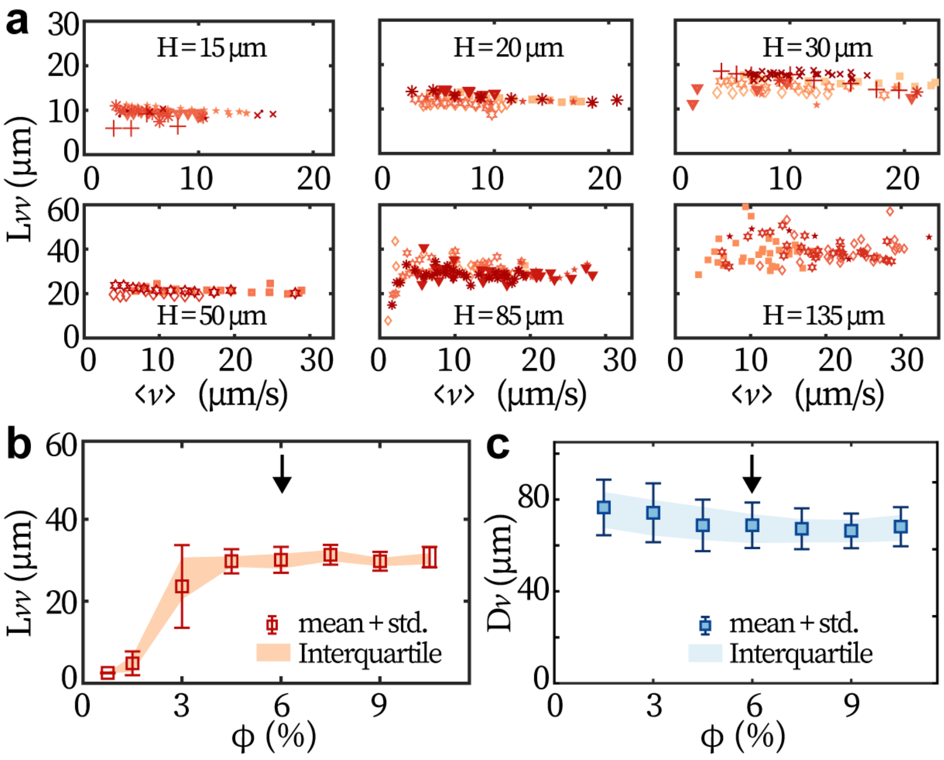

Figure S4: **Independence of vortex structure on activity and bacterial density** (a) Measurements of L_{vv} at different bacterial activity $\langle v \rangle$. Different symbols represent different experiments. Measurements are perform at $d_c = H/2$. (b) L_{vv} as a function of bacterial volume fraction ϕ (H=85µm, taken at $d_c = H/2$). (c) D_v as a function of ϕ. Black arrows mark the bacterial density used for the presented data in the main text.

S2 Critical heights measured in different representations.

The critical heights H_0 and H_1 manifest both in the characteristic lengths of the turbulence against H (L_{vv} and D_v) and in the kinetic energy spectra. Here we show that the measured values match to each other. H_0 and H_1 measured in different representations are displayed in Fig. S5a and b respectively. The x-axis of Fig. S5a shows H_0 measured as the x-intercept of the linear fitting to the initial fast-increasing trend in L_{vv}; while the y-axis is H_0 measured as where γ_2 drops below -2 in the energy spectra. The x-axis of Fig. S5b reports H_1 as where $H = D_v$ (with linear interpolation). The y-axis reports H_1 measured in energy spectra as where γ_1 drops below 1. Data gather around the identity lines ($y = x$, gray lines), showing that the two approaches are equivalent.

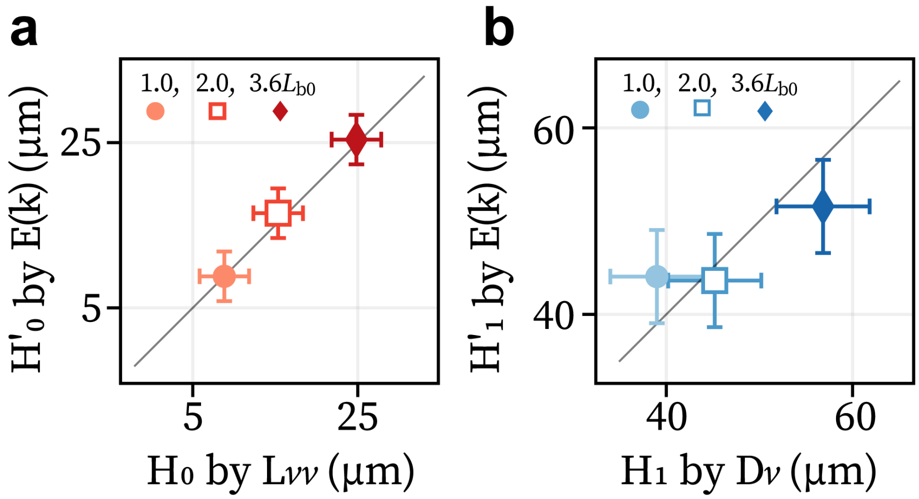

Figure S5: **Critical heights extracted from velocity correlation functions and from energy spectra**. H_0 (a) and H_1 (b) measured in different representations. The solid lines are the identity line ($y = x$). The error bars in the x and y axes represent the uncertainties in H.

S3 Measuring vertical flow using enhanced particle tracking velocimetry.

To confirm the velocity field has become isotropic at $H = H_1$, we measure the z-component of speed u_z in the following way. We first develop an enhanced particle tracking velocimetry (PTV) that extracts a bead's z-axis position by its appearance. The scheme of the technique is displayed in Fig. S6a-c. Beads are tracked with the TrackMate plugin of ImageJ and the algorithm primarily depends on fitting the beads' interior with a Laplacian-of-Gaussian (LoG) kernel. We exploit the mean pixel intensity I of the beads' interior and the quality factor Q which measures how well the LoG kernel fits. Fig. S6a-b display how the normalized metrics ($\tilde{I}$ and $\tilde{Q}$) varies with the focal plane height (Z_f, defined as shown in the schematic drawing). $\tilde{I}$ saturates at ~1 when $Z_f \leq 0$ and drops for $Z_f > 0$, i.e. the bead center turning from white to black. On the other hand, $\tilde{Q}$ peaks at $Z_f = 0$, i.e. the bead that is in-focus has the highest quality factor. These two trends are illustrated by the images of beads at the top of Fig. S6a. Combine these two metrics as $\tilde{Q} - 2\tilde{I}$, we obtain a metric that translates linearly to Z_f, see Fig. S6c.

Practically, we employ polystyrene beads of $a = 6$ µm diameter for PTV. The relationship between $\tilde{Q} - 2\tilde{I}$ and Z_f is calibrated by ~ 50 beads per sample after the bacterial suspension has ceased moving. Such configuration allows us to track all three coordinates of the beads in a slice of ~ $2a = 12$ µm thick. When a beads goes out of this slice, a track is considered finished. We pool $2 \times 10^3 - 1 \times 10^4$ tracks per sample. All tracks are broken down as 1 s long segments. This duration for each segment is determined by the characteristic time scale of the temporal velocity auto-correlation (1-3 s). The statistics of the speed (total arc length/1 s) in the z-axis and that in the xy-plane are displayed in the insets of Fig. S6d. Altogether, the mean or median of u_z/u_{xy} converges to $\sqrt{2}$ when $H = 40 - 50$ µm, which matches with H_1 observed from the vortex size (Fig. 2c, main text) or kinetic energy spectra (Fig. 3h, main text). The ratio supports that bacterial turbulence turns isotropic ($\langle u_x \rangle \approx \langle u_y \rangle \approx \langle u_z \rangle$).

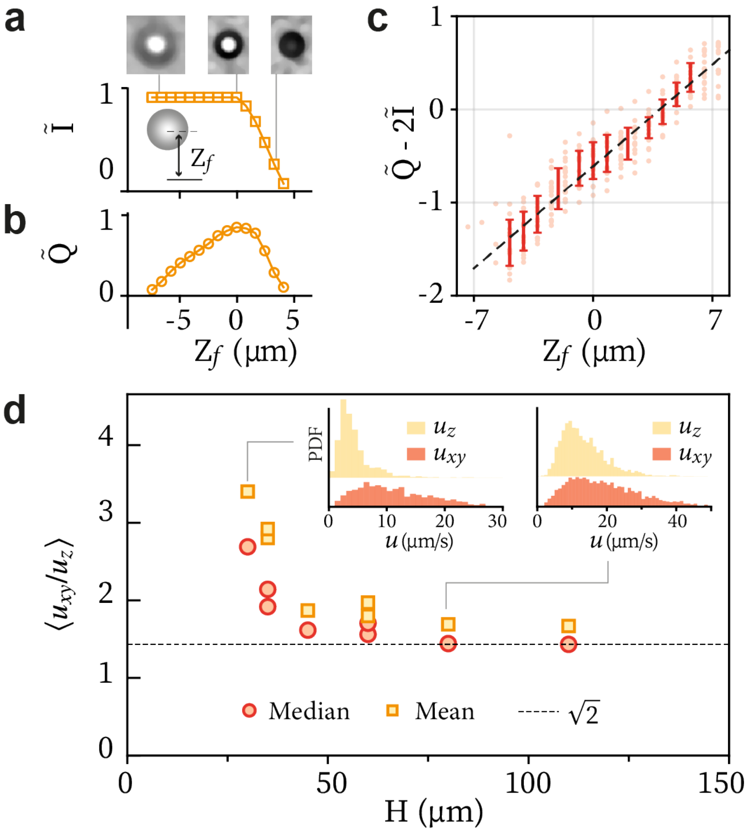

Figure S6: **Enhanced particle tracking velocimetry to measure 3D flows.** Scheme of extracting the z-position of beads in particle tracking velocimetry (PTV). (a) The normalized intensity ($\tilde{I}$) and (b) tracking quality factor ($\tilde{Q}$) of a fixed bead. Upper images display the beads at different relative heights Z_f. Insets: relative heights between the focal plane (solid line) and the bead, corresponding to the upper images respectively. (c) The composite metric $\tilde{Q}-2\tilde{I}$ corresponds linearly to the z-position. Dots: single beads; error bar: mean±std; dashed line: linear fit. (d) The mean ratio of the z-component of speed, u_z, to the xy-component of speed, u_{xy}. Insets: representative histograms of u_z and u_{xy} in samples of H = 30 μm (left) and 80 μm (right). Histograms are vertically shifted for clarity.

S4 Energy spectrum at low-k

Our theory predicts that the scaling $E(k) \sim k$ dominates the low-k end of the spectrum. To observe examine such spectral behavior for $H > 50$ μm, we measure samples with 10× magnification. Under this low magnification, we are able to extract the flow field in samples up to H=190 μm, see Fig. S7. The $E(k) \sim k$ scaling regime presents clearly while the spectra continue shifting to the left (Fig.3d-e, main text).

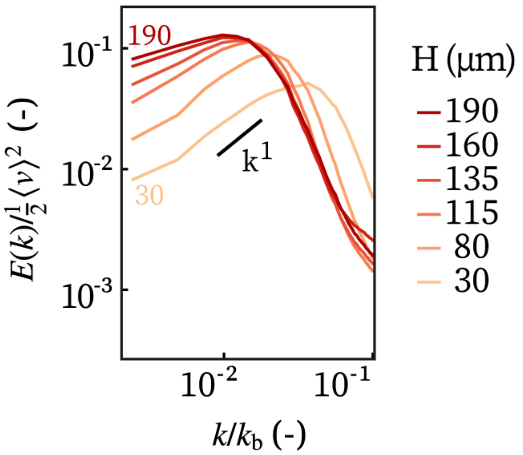

Figure S7: **Kinetic energy spectrum at the low-k end.** Flow fields acquired with lower microscope magnification (10×) are used to probe the spectral scaling at the low wavenumbers.

S5 Exponential distribution of vortex size

To validate one of theoretical assumptions that vortices in bacterial turbulence follow exponential size distributions, we measure vortex sizes with the algorithm used in Ref. (*2*). The algorithm first computes the Okubo-Weiss field $Q = -\partial_x u_x \cdot \partial_y u_y + \partial_x u_y \cdot \partial_y u_x$ from numerical data of the flow field. Furthermore, we locate vortex cores by scanning each frame (snapshot) of flow field with a square kernel of 10 μm size (~minimum size of vortex). Integrating the angular change of velocity vector along the kernel's boundary yields $\pm 2\pi$ if a vortex core is enclosed, or 0 otherwise. A region with $Q < 0$ and containing vortex core is considered as a vortex. Areas of such regions measure the vortex size projected in the xy-plane. We implement this algorithm in samples of varying H and confirm that the vortex size follows exponential distributions, see Fig. S8.

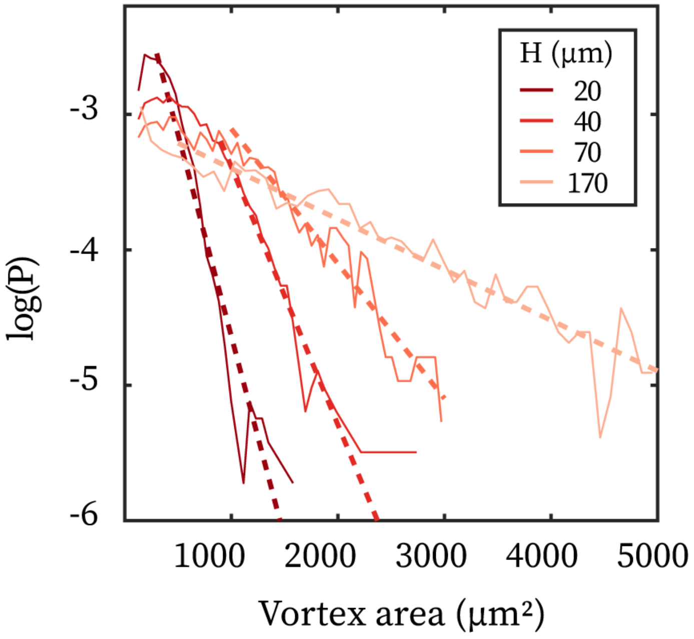

Figure S8: **Exponential distribution of vortex sizes in samples of different heights.** Cross-sectional sizes of the vortices in the xy-plane are extracted with the algorithm described in Ref. (*2*). Dashed lines are the exponential fits.

S6 Asymptotic scaling behaviors of the energy spectra

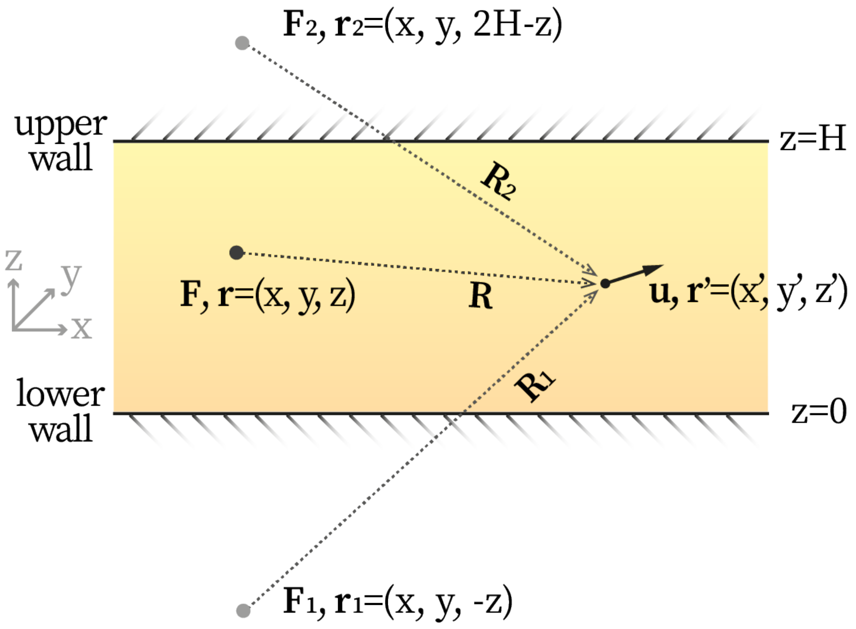

Figure S9: Scheme of the model

In order to understand the kinetic energy spectrum of the observed bacterial turbulence, we build a model based on the bacteria-fluid interactions. The model considers the dense bacteria suspension as a continuous medium of force monopoles $\boldsymbol{F}(x, y, z)$ and represents the effects of the upper wall $(z = H)$ and the lower wall $(z = 0)$ by two images $\boldsymbol{F}_1(x, y, 2H - z)$ and $\boldsymbol{F}_2(x, y, -z)$. Let $\boldsymbol{r}' = (x', y', z')$ be the location of interest and

$$\begin{cases} \boldsymbol{R} & = \boldsymbol{r}' - \boldsymbol{r}, \\ \boldsymbol{R}_1 & = \boldsymbol{r}' - \boldsymbol{r}_1, \\ \boldsymbol{R}_2 & = \boldsymbol{r}' - \boldsymbol{r}_2. \end{cases} \tag{S1}$$

The corresponding distances are:

$$\begin{cases} d = |\boldsymbol{R}| = \sqrt{(x' - x)^2 + (y' - y)^2 + (z' - z)^2}, \\ d_1 = |\boldsymbol{R}_1| = \sqrt{(x' - x)^2 + (y' - y)^2 + (z' + z)^2}, \\ d_2 = |\boldsymbol{R}_2| = \sqrt{(x' - x)^2 + (y' - y)^2 + (z' + z - 2H)^2}. \end{cases} \tag{S2}$$

We now write the flow velocity field $\boldsymbol{u}(\boldsymbol{r}')$ in terms of the forces analytically, $\boldsymbol{u}(\boldsymbol{r}') = \boldsymbol{F}(\boldsymbol{r}) \cdot \boldsymbol{G}(\boldsymbol{r}; \boldsymbol{r}')$, where $\boldsymbol{G}(\boldsymbol{r}; \boldsymbol{r}')$ is the Green function of the Stokes equations with two

walls. We approximate $\boldsymbol{G}(\boldsymbol{r};\boldsymbol{r}')$ by taking the first two image reflections for accounting the confining effects of the double walls (*3*):

$$\begin{aligned} G_{\alpha\beta} = \frac{1}{8\pi\mu}\Big\{ & \underbrace{\Big(\frac{\delta_{\alpha\beta}}{d} + \frac{d_\alpha d_\beta}{d^3}\Big)}_{\text{(i)}} - \underbrace{\Big(\frac{\delta_{\alpha\beta}}{d_1} + \frac{d_{1,\alpha} d_{1,\beta}}{d_1^3}\Big)}_{\text{(ii)}} - \underbrace{\Big(\frac{\delta_{\alpha\beta}}{d_2} + \frac{d_{2,i} d_{2,\beta}}{d_2^3}\Big)}_{\text{(iii)}} \\ & + \underbrace{2zz'\frac{\partial^2}{\partial d_{1,\alpha}\partial d_{1,\beta}}\frac{1}{d_1}}_{\text{(iv)}} + \underbrace{2(H-z)(H-z')\frac{\partial^2}{\partial d_{2,\alpha}\partial d_{2,\beta}}\frac{1}{d_2}}_{\text{(v)}} \Big\} \end{aligned} \tag{S3}$$

Here $\alpha, \beta = x, y$. As the velocity are only observed within the xy-plane, we focus on the velocity components u_α. In addition, we assume that the tensor components ($G_{3\alpha}$) are negligible and hence we only focus on the tensor components $G_{\alpha\beta}$. Note that this Green function does not satisfy the no-slip boundary condition at the two walls. However, the residue velocity is considered small enough to serve as a tangible approximation in order to assess the problem analytically.

Now we write the tensor in Fourier space (k_x, k_y, k_z). In the following part, we let $\boldsymbol{k} = (k_x, k_y)$ and $k = |\boldsymbol{k}|$; $\boldsymbol{R}_p$ be the xy-plane projection of $\boldsymbol{R}$, $\boldsymbol{R}_1$, and $\boldsymbol{R}_2$ (superscripts are removed as they are the same). Integrating the Fourier representation of terms (i-v) over the k_z axis and using the Residue theorem, we obtain (*4–6*):

$$\begin{aligned} (i) &= \frac{1}{\pi}\int d^2\boldsymbol{k}\ e^{i\boldsymbol{k}\cdot\boldsymbol{R}_p}\ e^{-k|z'-z|}\cdot\left(\frac{\delta_{\alpha\beta}}{k} - \frac{k_\alpha k_\beta(k|z'-z|+1)}{2k^3}\right) \\ (ii) &= \frac{1}{\pi}\int d^2\boldsymbol{k}\ e^{i\boldsymbol{k}\cdot\boldsymbol{R}_p}\ e^{-k|z'+z|}\cdot\left(\frac{\delta_{\alpha\beta}}{k} - \frac{k_\alpha k_\beta(k|z'+z|+1)}{2k^3}\right) \\ (iii) &= \frac{1}{\pi}\int d^2\boldsymbol{k}\ e^{i\boldsymbol{k}\cdot\boldsymbol{R}_p}\ e^{-k|2H-z'-z|}\cdot\left(\frac{\delta_{\alpha\beta}}{k} - \frac{k_\alpha k_\beta(k|2H-z'-z|+1)}{2k^3}\right) \\ (iv) &= -\frac{zz'}{\pi}\int d^2\boldsymbol{k}\ e^{i\boldsymbol{k}\cdot\boldsymbol{R}_p}\ e^{-k|z'+z|}k_\alpha k_\beta\cdot\frac{1}{k} \\ (v) &= -\frac{(H-z)(H-z')}{\pi}\int d^2\boldsymbol{k}\ e^{i\boldsymbol{k}\cdot\boldsymbol{R}_p}\ e^{-k|2H-z'-z|}k_\alpha k_\beta\cdot\frac{1}{k}. \end{aligned} \tag{S4}$$

With Eq. (S4), the Fourier representation of the Green function, $G_{\alpha\beta}$ reads:

$$\begin{aligned} G_{\alpha\beta} = \frac{1}{8\pi\mu}\cdot\frac{1}{\pi}\int d^2\boldsymbol{k}\ \frac{e^{i\boldsymbol{k}\cdot\boldsymbol{R}_p}}{k}\Bigg\{ & \underbrace{\Big[\delta_{\alpha\beta} - \frac{k_\alpha k_\beta(k|z'-z|+1)}{2k^2}\Big]\cdot e^{-k|z'-z|}}_{\text{(i')}} - \underbrace{\Big[\delta_{\alpha\beta} - \frac{k_\alpha k_\beta(k|z'+z|+1)}{2k^2}\Big]\cdot e^{-k|z'+z|}}_{\text{(ii')}} \\ & - \underbrace{\Big[\delta_{\alpha\beta} - \frac{k_\alpha k_\beta(k|2H-z'-z|+1)}{2k^2}\Big]\cdot e^{-k|2H-z'-z|}}_{\text{(iii')}} - \underbrace{zz'k_\alpha k_\beta e^{-k|z'+z|}}_{\text{(iv')}} - \underbrace{(H-z)(H-z')k_\alpha k_\beta e^{-k|2H-z'-z|}}_{\text{(v')}} \Bigg\} \end{aligned} \tag{S5}$$

Assuming that the active force generation does not depend on z, that is, $\boldsymbol{F} = \boldsymbol{F}(x, y)$, the flow velocity at the center of the chamber ($z' = H/2$) reads:

$$\begin{aligned} u_\alpha(x', y', z' = H/2) &= \int d^3\boldsymbol{r}\, G_{\alpha\beta}(x, x', y, y', z, z' = H/2) F_\beta(x, y) \\ &= \int d^2\boldsymbol{r}\, F_\beta(x, y) \int_0^H dz\, G_{\alpha\beta}(x, x', y, y', z, z' = H/2) \\ &= \frac{1}{8\pi^2\mu} \int d^2\boldsymbol{r}\, F_\beta(x, y) \cdot \underbrace{\int d^2\boldsymbol{k}\ \frac{e^{i\boldsymbol{k}\cdot\boldsymbol{R}_p}}{k} \cdot \int_0^H dz\, \big[(i') + ...(v')\big]}_{\overline{G}_{\alpha\beta}} \end{aligned} \tag{S6}$$

Denote the integrals of terms (i'-v') over z as (i"-v"). Sequentially, we obtain:

$$\begin{aligned} (\text{i"}) &= \int_0^H (\delta_{\alpha\beta} - \frac{k_\alpha k_\beta}{2k^2}) e^{-k|z'-z|} dz - \int_0^H \frac{k_\alpha k_\beta}{2k} |z - z'| e^{-k|z'-z|} dz \\ &= \delta_{\alpha\beta} \frac{2}{k}(1 - e^{-kH/2}) - \frac{k_\alpha k_\beta}{2k^3}(4 - 4e^{-kH/2} - kHe^{-kH/2}) \end{aligned} \tag{S7}$$

The asymptotic behaviors of Eq. (S7) are:

$$(\text{i"}) = \begin{cases} (\delta_{\alpha\beta} - \dfrac{k_\alpha k_\beta}{2k^2}) \cdot H, & \text{for } kH \ll 1; \\ (\delta_{\alpha\beta} - \dfrac{k_\alpha k_\beta}{k^2}) \cdot \dfrac{2}{k}, & \text{for } kH \gg 1 \end{cases} \tag{S8}$$

Similarly, the asymptotic behaviors of (ii") and (iii") are:

$$(\text{ii"}),\ (\text{iii"}) = \begin{cases} (\delta_{\alpha\beta} - \dfrac{k_\alpha k_\beta}{2k^2}) \cdot H, & \text{for } kH \ll 1; \\ 0. & \text{for } kH \gg 1 \end{cases} \tag{S9}$$

The rest of the terms read:

$$\begin{aligned} (\text{iv"}),\ (\text{v"}) &= \int_0^H z z' k_\alpha k_\beta e^{-k|z'+z|} dz \text{ or } \int_0^H (H - z)(H - z') k_\alpha k_\beta e^{-k|z'+z|} dz \\ &= k_\alpha k_\beta \frac{H}{2k^2} e^{-3kH/2}(e^{kH} - 1 - kH) \\ &= \begin{cases} \dfrac{k_\alpha k_\beta H^3}{4}, & \text{for } kH \ll 1; \\ 0, & \text{for } kH \gg 1 \end{cases} \end{aligned} \tag{S10}$$

Note that when $kH \ll 1$,

$$\frac{k_\alpha k_\beta H^3}{4} \ll (\delta_{\alpha\beta} - \frac{k_\alpha k_\beta}{2k^2}) \cdot H,$$

that is, (iv"),(v")≪(i"),(ii"),(iii"). Therefore, adding the terms above, the asymptotic behaviors of $\bar{G}_{\alpha\beta}$ (defined as shown in Eq. (S6)) is:

$$\bar{G}_{\alpha\beta} = \begin{cases} \int d^2\boldsymbol{k} \, \frac{e^{i\boldsymbol{k}\cdot\boldsymbol{R}_p}}{k} \left\{ (\delta_{\alpha\beta} - \frac{k_\alpha k_\beta}{2k^2}) \cdot (-H) \right\}, & \text{for } kH \ll 1; \\ \int d^2\boldsymbol{k} \, \frac{e^{i\boldsymbol{k}\cdot\boldsymbol{R}_p}}{k} \left\{ (\delta_{\alpha\beta} - \frac{k_\alpha k_\beta}{k^2}) \cdot \frac{2}{k} \right\}, & \text{for } kH \gg 1 \end{cases} \tag{S11}$$

Now we compute kinetic energy of the bacterial turbulent field, $\langle|\hat{\boldsymbol{u}}(\boldsymbol{k})|^2\rangle$, from which one obtains the spectrum density $E(k) \sim k\langle|\hat{\boldsymbol{u}}(\boldsymbol{k})|^2\rangle$ ($\langle\cdot\rangle$ means average over space and time):

$$\langle|\hat{\boldsymbol{u}}(\boldsymbol{k})|^2\rangle = \langle \bar{G}_{\alpha\beta}F_\beta \bar{G}_{\alpha\gamma}F^*_\gamma\rangle = \bar{G}_{\alpha\beta}\bar{G}_{\alpha\gamma}\langle F_\beta F^*_\gamma\rangle. \tag{S12}$$

For $kH \ll 1$, substituting Eq. (S11) into Eq. (S12):

$$\begin{aligned} \bar{G}_{\alpha\beta}\bar{G}_{\alpha\gamma}\langle F_\beta F^*_\gamma\rangle &\sim (\delta_{\alpha\beta} - \frac{k_\alpha k_\beta}{2k^2})(\delta_{\alpha\gamma} - \frac{k_\alpha k_\gamma}{2k^2}) \cdot (\frac{-H}{k})^2 \cdot \langle F_\beta F^*_\gamma\rangle \\ &= \left[\langle F_\alpha F^*_\alpha\rangle - \frac{3k_\alpha k_\beta}{4k^2}\langle F_\alpha F^*_\beta\rangle \right] \cdot (\frac{H}{k})^2 \\ &= \left[\frac{1}{4}\left(\langle F_x F^*_x\rangle + \langle F_y F^*_y\rangle \right) + \right. \\ &\left. \frac{3}{4k^2}\langle k_y^2 F_x^2 + k_x^2 F_y^2 - k_x k_y F_x F^*_y - k_x k_y F^*_x F_y\rangle \right] \cdot (\frac{H}{k})^2 \\ &= \frac{H^2}{4k^2}\langle F^2(\boldsymbol{k})\rangle + \frac{3H^2\eta^2}{4}\langle \omega^2(\boldsymbol{k})\rangle \end{aligned} \tag{S13}$$

Here η is the shear viscosity of the bacterial suspension, $\omega = \tilde{\boldsymbol{z}} \cdot \nabla \times \boldsymbol{u}$ is the vorticity component on the z axis. We have leveraged the relation: $\eta^2 k^4 \langle\omega^2(\boldsymbol{k})\rangle = \langle k_y^2 F_x^2 + k_x^2 F_y^2 - k_x k_y F_x F^*_y - k_x k_y F^*_x F_y\rangle$ to obtain this closed form for kinetic energy. This relation can be obtained by taking the curl of the Stokes equation, and represent the equation as a Poisson equation describing the diffusion of the vorticity. For details please see Ref. (*7*). Hence, $E(k)$ reads:

$$E(k) \sim k\langle|\hat{\boldsymbol{u}}(\boldsymbol{k})|^2\rangle \sim \frac{H^2}{4k}\langle F^2(\boldsymbol{k})\rangle + \frac{3H^2\eta^2 k}{4}\langle\omega^2(\boldsymbol{k})\rangle \tag{S14}$$

Similarly, for $kH \gg 1$:

$$\begin{aligned}\bar{G}_{\alpha\beta}\bar{G}_{\alpha\gamma}\langle F_\beta F_\gamma\rangle &\sim (\delta_{\alpha\beta}-\frac{k_\alpha k_\beta}{k^2})(\delta_{\alpha\gamma}-\frac{k_\alpha k_\gamma}{k^2})\cdot\frac{1}{k^4}\cdot\langle F_\beta F_\gamma^*\rangle\\ &=\left[\langle F_\alpha F_\alpha^*\rangle-\frac{k_\alpha k_\beta}{k^2}\langle F_\alpha F_\beta^*\rangle\right]\cdot\frac{1}{k^4}\\ &=\left[\langle k_y^2F_x^2+k_x^2F_y^2-k_xk_yF_xF_y^*-k_xk_yF_yF_x^*\rangle\right]\cdot\frac{1}{k^6}\\ &=\frac{\eta^2\langle\omega^2(\boldsymbol{k})\rangle}{k^2}\end{aligned}\tag{S15}$$

And the scaling of the energy spectrum is:

$$E(k)\sim\frac{\langle\omega^2(\boldsymbol{k})\rangle}{k}\tag{S16}$$

Under the assumption that $\langle F^2\rangle \ll \eta^2|\boldsymbol{k}|^2\langle\omega^2\rangle$, we have:

$$E(k)\sim\begin{cases}k\langle\omega^2(\boldsymbol{k})\rangle, & \text{for } kH\ll 1;\\ k^{-1}\langle\omega^2(\boldsymbol{k})\rangle, & \text{for } kH\gg 1\end{cases}\tag{S17}$$

It is therefore obvious that the scaling of the kinetic energy spectrum depends largely on the enstrophy spectrum. We now perform scaling analysis of $\langle\omega^2(\boldsymbol{k})\rangle$ on k.

We follow the treatment illustrated in Ref. (*2*). The main assumptions are:

1. **Uncorrelated 2D vortices**. The flow field is a superposition of uncorrelated vortices (numbered i, with a radius of R_i), whose major velocity components are in the xy plane (confined in the z axis).

2. **Vortex density distributes exponentially with respect to vortex area**. The spatial density of vortices of a given area ($n(a)$ with a the vortex area) follows an exponential distribution over a particular range: $n(a)=(N/Z)\cdot e^{-a/a^*}$ for $a_{\min}<a<a_{\max}$. $a_{\min}$ and $a_{\max}$ define an active range, where the vortices form directly due to mechanical work performed by the active stresses. Note that, other vortices may form due to shear between the active vortices. Also, a^* represents a characteristic area of active vortices, and it can be readily set as $a_{\min}$ (*2*). $N=\int_{a_{\min}}^{a_{\max}} da\, n(a)$ stands for the total number of vortices and $A=\int_{a_{\min}}^{a_{\max}} da e^{-a/a^*}=a^*(-e^{-a_{\min}/a^*}-e^{-a_{\max}/a^*})$.

3. **Step-function-like vorticity profile**. An active vortex has the Rakine vortex profile: its vorticity magnitude $\omega_i=\tilde{\boldsymbol{z}}\cdot\boldsymbol{\omega}_i=\omega_{v,i}f(r/R_i)$, with r the distance to the vortex center, $\omega_{v,i}$ a constant that can be different across vortices, and $f(r/R_i)$ the structure function with $f(r/R_i)=1$ for $r<R_i$ and $f(r/R_i)=0$ otherwise.

To analyze the spectral density of $\langle\omega^2(\boldsymbol{k})\rangle$, we first write the power spectrum of the vorticity magnitude field ($\omega(\boldsymbol{r})$) in Fourier space:

$$\omega^2(r)=\hat{\omega}^2(k)=\sum_{i,j}e^{i\boldsymbol{k}\cdot(\boldsymbol{r}_i-\boldsymbol{r}_j)}\omega_{v,i}\omega_{v,j}R_i^2R_j^2\hat{F}(kR_i)\hat{F}(kR_j).\tag{S18}$$

Here $\hat{F}(kR) = \frac{1}{R^2}\hat{f}(k)$ is a dimensionless structure vortex factor, with $\hat{f}(k)$ the Fourier representation of the vorticity structure function $f(r/R)$:

$$\hat{f}(k) = \int \frac{d^2r}{(2\pi)^2} e^{-i\boldsymbol{k}\cdot\boldsymbol{r}} f(\frac{r}{R}) = \frac{1}{2\pi}\int_0^\infty drrf(\frac{r}{R})J_0(kr), \tag{S19}$$

with J_0 a Bessel function of the first kind. By substituting $\zeta = r/R$ into Eq. (S19), $\hat{F}(kR)$ reads:

$$\hat{F}(kR) = \frac{1}{2\pi}\int_0^\infty d\zeta\zeta f(\zeta)J_0(k\zeta R) \tag{S20}$$

When the vortices do not correlate spatially, only the diagonal terms in the sum would survive upon averaging. Then,

$$\langle\omega^2(k)\rangle = \sum_i \omega_{v,i}^2 R_i^4 \hat{F}^2(kR_i) \approx \int_{R_{\min}}^{R_{\max}} dRn(R)\omega_v^2(R)R^4\hat{F}^2(kR), \tag{S21}$$

where the summation is approximated with integration over the vortex population.

With two observations by Ref. (*2*), that 1) vortices are circular and thus $a_i = \pi R_i^2$ and 2) all vortices have a uniform vorticity ω_v, we further represent Eq. (S21) with dimensionless wavelengths $\kappa = kR^*$ and $\xi = kR$:

$$\begin{aligned}\langle\omega^2(k)\rangle &\approx \int_{R_{\min}}^{R_{\max}} dRn(R)\omega_v^2(R)R^4\hat{F}^2(kR)\\ &= \frac{2\pi N\omega_v^2}{A}\int_{\xi_{\min}}^{\xi_{\max}} d(\frac{\xi}{k})e^{-\xi^2/\kappa^2}\left(\frac{\xi}{k}\right)^5\hat{F}^2(\xi)\\ &= \frac{2\pi N\omega_v^2}{Ak^6}\int_{\xi_{\min}}^{\xi_{\max}} d\xi e^{-\xi^2/\kappa^2}\xi^5\hat{F}^2(\xi)\end{aligned} \tag{S22}$$

Note that the second assumption (assumption of exponentially-distributed vortices) used for the above derivation, which entails $n(R) = |da/dR|n(a) = 2\pi RN/Ze^{-R^2/R^{*2}}$. Finally, we exploit the third assumption that the vortices have a Rankine vortex structure, which helps give an explicit expression of $\hat{F}(kR)$. Bring $f(\zeta) = 1$ for $\zeta < 1$ and 0 otherwise) into Eq. (S20):

$$\hat{F}(\xi) = \frac{1}{2\pi}\int_0^1 d\zeta\zeta J_0(\xi\zeta) \quad = \frac{1}{2\pi\xi}J_1(\xi). \tag{S23}$$

Eq. (S22) and Eq. (S23) together give:

$$\begin{aligned}\langle\omega^2(\kappa)\rangle &\approx \frac{2\pi N\omega_v^2}{Ak^6}\int_{\xi_{\min}}^{\xi_{\max}} d\xi e^{-\xi^2/\kappa^2}\xi^3\frac{1}{4\pi^2}J_1^2(\xi)\\ &= \frac{N\omega_v^2 R^{*6}}{8\pi A}e^{-\kappa^2/2}\big[I_0(\frac{\kappa^2}{2}) - I_1(\frac{\kappa^2}{2})\big]\end{aligned} \tag{S24}$$

The asymptotic behavior of Eq. (S24) is:

$$\langle\omega^2(k)\rangle \sim \begin{cases} k^0, & \text{for } kR^* \ll 1;\\ k^{-3}, & \text{for } kR^* \gg 1,\end{cases} \tag{S25}$$

with $k^* = 2\pi/R^*$.

Finally, the scaling of the kinetic energy spectrum, which results from the competition kH and kR^*, are:

$$E(k) \sim \begin{cases} k^{-1} & \text{for } kR^* \ll 1 \text{ and } kH \gg 1, \\ k^{-4} & \text{for } kR^* \gg 1 \text{ and } kH \gg 1, \\ k^{1} & \text{for } kR^* \ll 1 \text{ and } kH \ll 1, \\ k^{-2} & \text{for } kR^* \gg 1 \text{ and } kH \ll 1. \end{cases} \tag{S26}$$

S7 Inherent vortex sizes analyzed by kinetic theory

We employ kinetic theory to model the system and to resolve the relation between the vortex size and the confinement height. We follow similar approaches as developed for active nematics (*8–10*) and further include the self-propulsion of bacteria into the picture. Please note that this model is a separate effort from the previous hydrodynamic model we develop in Section. S6. Our goal here is to give a possible explanation to the $H^{0.5}$ scaling observed and to situate this finding within the context of prior theoretical efforts.

The scheme of the system is the same as shown in Fig. S9. Now the bacteria suspension between two parallel plates is considered as a continuum of force dipoles:

$$\boldsymbol{\Sigma}^p(\boldsymbol{r}) = \alpha\delta(\boldsymbol{r})\boldsymbol{pp}, \tag{S27}$$

Here α is the magnitude of a force dipole ($\alpha < 0$ for pushers and $\alpha > 0$ for pullers), δ is the Dirac delta-function, and $\boldsymbol{p}$ is the unit vector in the swimming direction of a single bacterium.

The system at time t is fully described by the probability distribution function $\psi(\boldsymbol{r},\boldsymbol{p},t)$ of finding a bacterium with center-of-mass position $\boldsymbol{r}$ and orientation $\boldsymbol{p}$. $\psi(\boldsymbol{r},\boldsymbol{p},t)$ satisfies $\int_V\int_\Omega \psi(\boldsymbol{r},\boldsymbol{p},t)d\boldsymbol{p}d\boldsymbol{r} = N$, where N is the total number of bacteria, V is the volume of the system, Ω is the unit sphere in the orientational space. The evolution of $\psi(\boldsymbol{r},\boldsymbol{p},t)$ is described by the Smoluchowski equation:

$$\partial_t\psi + \nabla\cdot(\dot{\boldsymbol{r}}\psi) + \nabla_p\cdot(\dot{\boldsymbol{p}}\psi) = 0, \tag{S28}$$

with $\nabla_p = (\boldsymbol{I}-\boldsymbol{pp})\cdot\frac{\partial}{\partial\boldsymbol{p}}$ the gradient operator on Ω.

The flux velocities of translation $\dot{\boldsymbol{r}}$ and rotational motions $\dot{\boldsymbol{p}}$ are given by:

$$\begin{cases}\dot{\boldsymbol{r}} = V_s\boldsymbol{p} + \boldsymbol{u} - D\nabla\ln\psi,\\ \dot{\boldsymbol{p}} = (\boldsymbol{I}-\boldsymbol{pp})\cdot(\beta\boldsymbol{E}+\boldsymbol{W})\cdot\boldsymbol{p} - d\nabla_p\ln\psi.\end{cases} \tag{S29}$$

V_s is the self-propelled swimming velocity, $\boldsymbol{u}(\boldsymbol{r},t)$ is the local background flow velocity. D and d denote the translational and rotational diffusion coefficients, respectively. $\boldsymbol{E} = \frac{1}{2}(\nabla\boldsymbol{u}+\nabla\boldsymbol{u}^T)$ and $\boldsymbol{W} = \frac{1}{2}(\nabla\boldsymbol{u}-\nabla\boldsymbol{u}^T)$ denote the rate-of-strain and vorticity tensors, respectively. $\beta = \frac{A^2-1}{A^2+1}$ characterizes the shape of the bacterium with A being the aspect ratio.

Next we write the background flow field $\boldsymbol{u}$ in terms of ψ. The system is in the low-Reynolds number regime and $\boldsymbol{u}$ is thus the single-bacterium flow field $\boldsymbol{u}^{(i)}(\boldsymbol{r},\boldsymbol{p})$ added up. The Stokes equation and the incompressibility condition for $\boldsymbol{u}^{(i)}(\boldsymbol{r},\boldsymbol{p})$ writes:

$$-\eta\nabla^2\boldsymbol{u}^{(i)}(\boldsymbol{r}) + \nabla P^{(i)}(\boldsymbol{r}) = \nabla\cdot\boldsymbol{\Sigma}^p, \qquad \nabla\cdot\boldsymbol{u}^{(i)}(\boldsymbol{r}) = 0 \tag{S30}$$

where $P^{(i)}(\boldsymbol{r})$ denotes the pressure field. Summing over all bacteria, we have:

$$-\eta\nabla^2\boldsymbol{u}(\boldsymbol{r},t) + \nabla P(\boldsymbol{r},t) = \nabla\cdot\int_V\int_\Omega \alpha\boldsymbol{pp}\delta(\boldsymbol{r}-\boldsymbol{r}')\psi(\boldsymbol{r}',\boldsymbol{p},t)d\boldsymbol{p}d\boldsymbol{r}', \qquad \nabla\cdot\boldsymbol{u}(\boldsymbol{r},t) = 0. \tag{S31}$$

where $\int_V\int_\Omega \alpha\boldsymbol{pp}d\boldsymbol{p}$ is equivalently the stress tensor in the Stokes equation, which allows us to define the traceless active stress $\Sigma(\boldsymbol{r},t) = \int_V\int_\Omega \alpha(\boldsymbol{pp}-\frac{1}{3}\boldsymbol{I})d\boldsymbol{p}$.

We perform linear stability analyzes on the Smoluchowski equation Eq. (S28). To facilitate direct physical interpretation, we first detail the the system's evolution into the evolution of the zeroth, first, and the second moments of $\boldsymbol{p}$, which are respectively the local bacterial concentration $c(\boldsymbol{r},t)$, the polarity $\boldsymbol{n}(\boldsymbol{r},t)$, and the orientational order $\boldsymbol{Q}(\boldsymbol{r},t)$:

$$\begin{cases} c(\boldsymbol{r},t) = \int_\Omega \psi(\boldsymbol{r},\boldsymbol{p},t)d\boldsymbol{p}, \\ \boldsymbol{n}(\boldsymbol{r},t) = \frac{1}{c(\boldsymbol{r},t)}\int_\Omega \boldsymbol{p}\psi(\boldsymbol{r},\boldsymbol{p},t)d\boldsymbol{p}, \\ \boldsymbol{Q}(\boldsymbol{r},t) = \frac{1}{c(\boldsymbol{r},t)}\int_\Omega (\boldsymbol{pp}-\frac{1}{3}\boldsymbol{I})\psi(\boldsymbol{r},\boldsymbol{p},t)d\boldsymbol{p}, \end{cases} \tag{S32}$$

We write Eq. (S28) in terms of c, $\boldsymbol{n}$, and $\boldsymbol{Q}$ and investigate the stability of the aligned state, similarly as in Ref. (*8*).

The aligned initial state entails that all bacteria are aligned in the $\hat{\boldsymbol{x}}$ direction at $t=0$, with a uniform concentration $c_0 = N/V$:

$$\psi(\boldsymbol{r},\boldsymbol{p},0) = c_0\delta(\boldsymbol{p}-\hat{\boldsymbol{x}}). \tag{S33}$$

Thereafter, the system stays locally aligned ($\frac{\eta}{c_0\alpha} \ll \frac{1}{d}$) and evolves as:

$$\psi(\boldsymbol{r},\boldsymbol{p},t) = c(\boldsymbol{r},t)\delta[\boldsymbol{p}-\boldsymbol{n}(\boldsymbol{r},t)]. \tag{S34}$$

Under such conditions, the magnitude of orientational order is fixed to $Q_0 = 1$. Due to the uniaxial symmetry, $\boldsymbol{Q}$ is now completely determined by $\boldsymbol{n}$ as $\boldsymbol{Q} = Q_0(\boldsymbol{nn}-\frac{1}{3}\boldsymbol{I}) = \boldsymbol{nn}-\frac{1}{3}\boldsymbol{I}$. We thus truncate the moment equations at the first order.

We now add perturbation to each dynamic variable (c, $\boldsymbol{n},\boldsymbol{u}$) and analyze the growth rate of its modes in the wavenumber space. Let $\mathcal{V}$ denote an arbitrary dynamic variable and its temporal evolution is $\mathcal{V}(t) = \mathcal{V}(0)+\delta\mathcal{V}(t)$. In the Fourier space, $\delta\mathcal{V} = \int \delta\tilde{\mathcal{V}}e^{i\boldsymbol{k}\cdot\boldsymbol{r}+\sigma t}d\boldsymbol{k}$, with $\tilde{\mathcal{V}}$ the amplitude of the Fourier modes and σ the corresponding growth rates. Additionally, we have $\delta\boldsymbol{n}\cdot\hat{\boldsymbol{x}} = 0$ to ensure that the length of $\boldsymbol{n}$ remains 1 up to the linear order. Altogether, the perturbed equations of the zeroth and first moments are:

$$\begin{cases} \partial_t\delta c = -V_s\nabla\delta c\cdot\hat{\boldsymbol{x}} - c_0V_s\nabla\cdot\delta\boldsymbol{n} + D\nabla^2\delta c, \\ \partial_t\delta\boldsymbol{n} = -V_s\nabla\cdot(\hat{\boldsymbol{x}}\delta\boldsymbol{n}) + D\nabla^2\delta\boldsymbol{n} + (\boldsymbol{I}-\hat{\boldsymbol{x}}\hat{\boldsymbol{x}})\cdot(\beta\delta\boldsymbol{E}+\delta\boldsymbol{W})\cdot\hat{\boldsymbol{x}}. \end{cases} \tag{S35}$$

Meanwhile, the perturbation on $\boldsymbol{u}$ in the Stokes equation and the incompressibility condition in the $\boldsymbol{k}$-space gives:

$$\delta\tilde{\boldsymbol{u}} = \frac{i\alpha}{\eta k^2}(\boldsymbol{I}-\hat{\boldsymbol{k}}\hat{\boldsymbol{k}})\cdot(c_0\delta\tilde{\boldsymbol{n}}\hat{\boldsymbol{x}} + c_0\hat{\boldsymbol{x}}\delta\tilde{\boldsymbol{n}} + \delta\tilde{c}\hat{\boldsymbol{x}}\hat{\boldsymbol{x}})\cdot\boldsymbol{k}. \tag{S36}$$

$k = |\boldsymbol{k}|$ and $\hat{\boldsymbol{k}} = \frac{\boldsymbol{k}}{k}$. The rate-of-strain and vorticity tensors in the Fourier space are: $\delta\tilde{E} = \frac{i}{2}(\delta\tilde{\boldsymbol{u}}\boldsymbol{k}+\boldsymbol{k}\delta\tilde{\boldsymbol{u}})$, $\delta\tilde{W} = \frac{i}{2}(\delta\tilde{\boldsymbol{u}}\boldsymbol{k}-\boldsymbol{k}\delta\tilde{\boldsymbol{u}})$. With this, Eq. (S35) become eigenvalue problems of $\delta\tilde{c}$ and $\delta\tilde{\boldsymbol{n}}$:

$$\begin{cases} (\sigma+Dk^2+iV_sk_x)\delta\tilde{c} = & -ic_0V_s\boldsymbol{k}\cdot\delta\tilde{\boldsymbol{n}}, \\ (\sigma+Dk^2+iV_sk_x)\delta\tilde{\boldsymbol{n}} = & -\frac{\alpha(\beta+1)}{2\eta k^2}[c_0k_x^2\delta\tilde{\boldsymbol{n}} - \frac{k_x}{k^2}(2c_0k_x\boldsymbol{k}\cdot\delta\tilde{\boldsymbol{n}}+k_x^2\delta\tilde{c})\boldsymbol{k}_\perp] \\ & -\frac{\alpha(\beta-1)}{2\eta k^2}[c_0\frac{1}{k^2}(k_\perp^2-k_x^2)\boldsymbol{k}\cdot\delta\tilde{\boldsymbol{n}} + \frac{k_x}{k^2}(k^2-k_x^2)\delta\tilde{c}]\boldsymbol{k}_\perp, \end{cases} \tag{S37}$$

with $\boldsymbol{k}_\perp = (0, k_y, k_z)$.

As $\delta\tilde{\boldsymbol{n}}\cdot\hat{x} = 0$ in the current setting, we can decompose the wave vector $\boldsymbol{k}$ into orthogonal bases $\frac{\delta\tilde{\boldsymbol{n}}}{|\delta\tilde{\boldsymbol{n}}|}$, $\hat{\boldsymbol{x}}$, and $\frac{\delta\tilde{\boldsymbol{n}}}{|\delta\tilde{\boldsymbol{n}}|}\times\hat{\boldsymbol{x}}$. Projections into these bases correspond respectively to the splay $\boldsymbol{k}\cdot\delta\tilde{\boldsymbol{n}}$, bend $\hat{\boldsymbol{x}}\times(\boldsymbol{k}\times\delta\tilde{\boldsymbol{n}})$, and twist $\hat{\boldsymbol{x}}\cdot(\boldsymbol{k}\times\delta\tilde{\boldsymbol{n}})$ deformations of the bacterial suspension.

One eigenmode of Eq. (S37) is obtained when $\boldsymbol{k}\cdot\delta\tilde{\boldsymbol{n}} = 0$, which means the absence of the splay mode and we thus denote this eigenmode the twist-bend mode. Its growth rate σ_{tb} is obtained from Eq. (S37) with $\delta\tilde{c} = 0$ and $\boldsymbol{k}\cdot\delta\tilde{\boldsymbol{n}} = 0$:

$$\sigma_{tb} = -\frac{c_0\alpha(\beta+1)}{2\eta k^2}k_x^2 - Dk^2 - iV_s k_x \tag{S38}$$

The other two eigenmodes are the splay-bend modes, that is, in the absence of twist deformations ($\hat{\boldsymbol{x}}\cdot(\boldsymbol{k}\times\delta\tilde{\boldsymbol{n}}) = 0$). In this case, the wave vector $\boldsymbol{k}$ lies in the $\hat{\boldsymbol{x}} - \delta\tilde{\boldsymbol{n}}$ plane and can therefore be represented in polar coordinates: $\boldsymbol{k} = k(\cos\theta\hat{\boldsymbol{x}} + \sin\theta\frac{\delta\tilde{\boldsymbol{n}}}{|\delta\tilde{\boldsymbol{n}}|})$, with θ is the angle between $\boldsymbol{k}$ and $\hat{\boldsymbol{x}}$. Eq. (S37) then becomes:

$$\begin{cases}(\sigma + Dk^2 + iV_s k_x)\delta\tilde{c} = -ic_0 k\sin\theta\delta\tilde{n},\\ (\sigma + Dk^2 + iV_s k_x)\delta\tilde{n} = -\frac{\alpha}{2\eta}[(\beta+1)\cos^2\theta - (\beta-1)\sin^2\theta](c_0\cos2\theta\delta\tilde{n} - \frac{1}{2}\sin2\theta\delta\tilde{c}).\end{cases} \tag{S39}$$

The growth rates of the two splay-bend modes $\sigma_{sb}^{\pm}$ are:

$$\sigma_{sb}^{\pm} = \frac{1}{2}f(\theta)\cos2\theta\left[1 \pm \sqrt{1 + 4iV_s k\frac{\sin^2\theta\cos\theta}{f(\theta)\cos^2 2\theta}}\right] - Dk^2 - iV_x k_x, \tag{S40}$$

with $f(\theta) = -\frac{c_0\alpha}{2\eta}[(\beta+1)\cos^2\theta - (\beta-1)\sin^2\theta]$.

What determines the characteristic vortex size in a given system is the fastest growing mode, corresponding to the largest positive growth rates in the wavenumber space. In Fig. S10a, we display the growth rates of these eigenmodes in the wavenumber space (dispersion relations).

When there is no confinement, in the long-wavelength limit ($\boldsymbol{k}\to\boldsymbol{0}$), the asymptotic dispersion relation is:

$$\sigma_{\text{free}} = -\frac{c_0\alpha(\beta+1)}{2\eta} - Dk^2. \tag{S41}$$

Here σ_{free} monotonically decreases with k, indicating the mode with the longest wavelength will dominate the system, which has been long known as the long-wavelength instability (*8*). Confinement in z qualitatively changes the picture. We illustrate this point with the dispersion relation of the twist-bend mode $\sigma_{tb}(k)$, which is representative for the other two modes. Due to confining boundary conditions at the walls, perturbations need to satisfy the standing wave condition: $|k_z| = n\pi/H$ ($n = 1, 2...$), and k_z now has an minimum positive value π/H. This causes the first term of Eq. (S38), which scales with k_x^2/k^2, to become 0 in the long-wavelength limit.

In Fig. S10a, we display the dispersion relations' asymptotic behaviors in the long-wavelength limit by varying k_x and taking $k_y = 0$ and a non-zero k_z. Note that varying k_y makes no qualitative change as σ_{tb} decreases monotonically with k_y, Eq. (S38). However,

varying the minimum value of k_z illustrates the categorical difference between a system primarily confined in one axis and a system whose confinement in all axes are symmetric. In Fig. S10b, we compare σ_{free} with σ_{tb} of varying k_z. At small k_z, σ_{tb} approaches σ_{free} but it pertains the same qualitative behavior ($\sigma_{tb} \to 0$) at the long-wavelength limit. Meanwhile, σ_{tb} under larger k_z (stronger confinement) appear to decrease in general. This trend indicates σ_{tb} will be nowhere positive above certain k_z, i.e., instability cannot emerge, see the lowest σ_{tb} in Fig. S10b. This critical k_z corresponds to a lower bound in H (H_0) for the onset of bacterial turbulence.

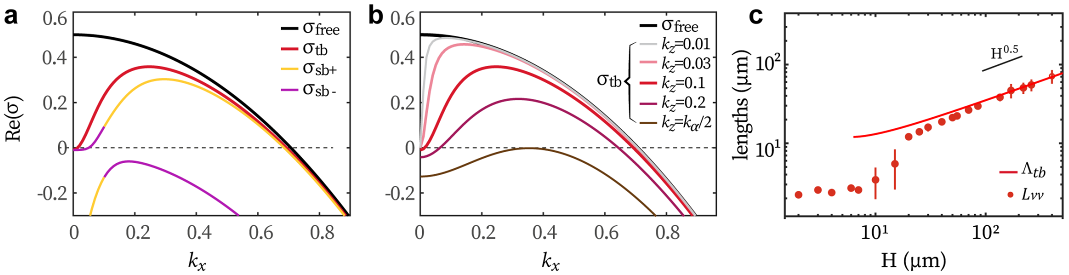

Figure S10: Growth rates of different eigenmodes in the wavenumber space. (a) $\frac{c_0\alpha}{\eta} = D = V_s = 1$, $\beta = 0$, $k_y = 0$ and $k_z = 0.1$. (b) dispersion of the twist-bend mode when system size increases (k_z decreases). (c) the dominant vortex size Λ for the twist-bend eigenmode as a function of the confinement size H, Eq.(S42).

Hence, the displayed dispersion relations reveal two inherent lengths (wavenumbers) of the system. The maximal k_z^0 that allows instability to emerge and the fastest-growing wavenumber of instability k_v. For convenience in notation, let k_α denote the wavenumber where $\sigma(k)_{\text{free}} = 0$. From Eq. (S41), we obtain $k_\alpha = \sqrt{-\frac{c_0\alpha(\beta+1)}{2D\eta}}$ for pusher ($\alpha < 0$). Now the wavenumber k_v of the highest growth rate satisfies $k_v^2 = k_\alpha k_z - k_z^2$, $k_z = \pi/H$ (under the minimum k_z and $k_y = 0$, Eq. (S38)). From the expression we see k_v is determined by the confinement. Let $\Lambda = 2\pi/k_v$ denote the dominant vortex size:

$$\Lambda = 2\left(\frac{2}{l_\alpha H} - \frac{1}{H^2}\right)^{-1/2}. \tag{S42}$$

When $H \gg l_\alpha$, $\Lambda \sim H^{0.5}$. This explains the experimental observations in Fig. 2a and Fig. 3g in the main text, see also Fig. S10c. Note that similar analysis also applies for the splay-bend mode and the dominant vortex is found to follow the same $H^{0.5}$ scaling.

Substituting the expression of k_v into Eq. (S38), the maximum growth rate $\sigma_{tb}^{\max} = Dk_\alpha(k_\alpha - 2k_Z)$. With l_α as the sole parameter, we fit the experimentally measured velocity correlation length L_{vv} in large confinement sizes ($H > H_1$) with Eq. (S42), see Fig. S10c. l_α is obtained to be 6 μm, in line with $l_\alpha = 2\pi\sqrt{-\frac{2D\eta}{c_0\alpha(\beta+1)}} \approx 9$ μm (using typical values $\alpha = -1.9$ μm ·0.42 pN (*11*) and $D = 50$ μm ^{2}/s (*12*)).

As an end note, our analysis of the aligned states take basis on $\frac{\eta}{c_0\alpha} \ll \frac{1}{d}$, which is essentially a quasi-steady approximation. Such approximation holds well in the bacterial system, where $1/d \sim 10$ s (*11, 13*) and $\eta/c_0\alpha \sim 0.04$ s (*11*).

References

1. Berke, A. P., Turner, L., Berg, H. C. & Lauga, E. Hydrodynamic attraction of swimming microorganisms by surfaces. *Phys. Rev. Lett.* **101**, 038102 (2008).

2. Giomi, L. Geometry and topology of turbulence in active nematics. *Phys. Rev. X* **5**, 031003 (2015).

3. Liron, N. & Mochon, S. Stokes flow for a stokeslet between two parallel flat plates. *J. Eng. Math.* **10**, 287–303 (1976).

4. Meng, F., Bennett, R. R., Uchida, N. & Golestanian, R. Conditions for metachronal coordination in arrays of model cilia. *Proc. Natl. Acad. Sci. U.S.A.* **118**, e2102828118 (2021).

5. Ishida, S., Yang, Y., Meng, F. & Matsunaga, D. Field-controlling patterns of sheared ferrofluid droplets. *Phys. Fluids* **34**, 063309 (2022).

6. Lisicki, M. Four approaches to hydrodynamic green's functions – the oseen tensors. *arXiv:1312.6231* (2013). URL `https://doi.org/10.48550/arXiv.1312.6231`. `1312.6231`.

7. Martínez-Prat, B. *et al.* Scaling regimes of active turbulence with external dissipation. *Phys. Rev. X* **11**, 031065 (2021).

8. Saintillan, D. & Shelley, M. J. Instabilities, pattern formation, and mixing in active suspensions. *Phys. Fluids* **20**, 123304 (2008).

9. Saintillan, D. & Shelley, M. J. *Theory of Active Suspensions*, chap. 9, 319–355 (Springer, New York, NY, 2015).

10. Chandrakar, P. *et al.* Confinement controls the bend instability of three-dimensional active liquid crystals. *Phys. Rev. Lett.* **125**, 257801 (2020).

11. Drescher, K., Dunkel, J., Cisneros, L. H., Ganguly, S. & Goldstein, R. E. Fluid dynamics and noise in bacterial cell–cell and cell–surface scattering. *Proc. Natl. Acad. Sci. U.S.A.* **108**, 10940–10945 (2011).

12. Koch, D. L. & Subramanian, G. Collective hydrodynamics of swimming microorganisms: Living fluids. *Annual Review of Fluid Mechanics* **43**, 637–659 (2011).

13. Huo, H., He, R., Zhang, R. & Yuan, J. Swimming escherichia coli cells explore the environment by lévy walk. *Appl. Environ. Microbiol.* **87**, e02429–20 (2021).

Movies captions

Movie S1. Representative bacterial turbulence in a sample of H = 10 µm. Left: the bright field video (contrast adjusted); right: the corresponding vorticity field with streamlines. The movie plays in real time. Scale bar: 50 μm.

Movie S2. Representative bacterial turbulence in a sample of H = 30 µm. Left: the bright field video (contrast adjusted); right: the corresponding vorticity field with streamlines. The movie plays in real time. Scale bar: 50 μm.

Movie S3. Representative bacterial turbulence in a sample of H = 170 µm. Left: the bright field video (contrast adjusted); right: the corresponding vorticity field with streamlines. The movie plays in real time. Scale bar: 50 μm.
